# Correlated Anion Disorder in Heteroanionic Cubic TiOF_2_

**DOI:** 10.1021/jacs.4c06304

**Published:** 2024-07-26

**Authors:** Christophe Legein, Benjamin J. Morgan, Alexander G. Squires, Monique Body, Wei Li, Mario Burbano, Mathieu Salanne, Thibault Charpentier, Olaf J. Borkiewicz, Damien Dambournet

**Affiliations:** † Institut des Molécules et des Matériaux du Mans (IMMM), UMR 6283 CNRS, Le Mans Université, Avenue Olivier Messiaen, 72085 Le Mans Cedex 9, France; ‡ Department of Chemistry, University of Bath, Claverton Down BA2 7AY, United Kingdom; § The Faraday Institution, Quad One, Harwell Science and Innovation Campus, Didcot OX11 0RA, United Kingdom; ∥ School of Chemistry, University of Birmingham, Edgbaston, Birmingham, B15 2TT, United Kingdom; ⊥ Sorbonne Université, CNRS, Physico-chimie des électrolytes et nano-systèmes interfaciaux, PHENIX, F-75005 Paris, France; # Réseau sur le Stockage Electrochimique de l’Energie (RS2E), FR CNRS 3459, 80039 Amiens Cedex, France; ∇ Université Paris-Saclay, CEA, CNRS, NIMBE, 91191 Gif-sur-Yvette Cedex, France; ⊗ X-ray Science Division, Advanced Photon Source, Argonne National Laboratory, Argonne, Illinois 60439, United States

## Abstract

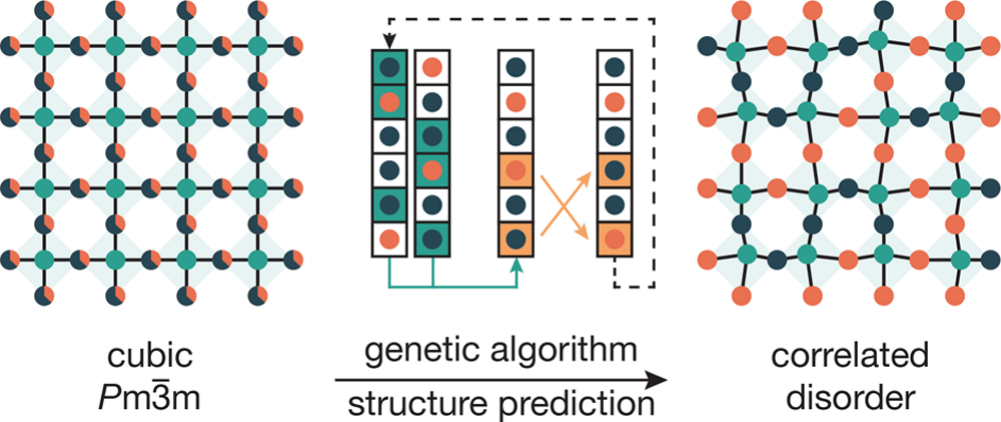

Resolving anion configurations in heteroanionic materials is crucial for understanding and controlling their properties. For anion-disordered oxyfluorides, conventional Bragg diffraction cannot fully resolve the anionic structure, necessitating alternative structure determination methods. We have investigated the anionic structure of anion-disordered cubic (ReO_3_-type) TiOF_2_ using X-ray pair distribution function (PDF), ^19^F MAS NMR analysis, density functional theory (DFT), cluster expansion modeling, and genetic-algorithm structure prediction. Our computational data predict short-range anion ordering in TiOF_2_, characterized by predominant *cis*-[O_2_F_4_] titanium coordination, resulting in correlated anion disorder at longer ranges. To validate our predictions, we generated partially disordered supercells using genetic-algorithm structure prediction and computed simulated X-ray PDF data and ^19^F MAS NMR spectra, which we compared directly to experimental data. To construct our simulated ^19^F NMR spectra, we derived new transformation functions for mapping calculated magnetic shieldings to predicted magnetic chemical shifts in titanium (oxy)fluorides, obtained by fitting DFT-calculated magnetic shieldings to previously published experimental chemical shift data for TiF_4_. We find good agreement between our simulated and experimental data, which supports our computationally predicted structural model and demonstrates the effectiveness of complementary experimental and computational techniques in resolving anionic structure in anion-disordered oxyfluorides. From additional DFT calculations, we predict that increasing anion disorder makes lithium intercalation more favorable by, on average, up to 2 eV, highlighting the significant effect of variations in short-range order on the intercalation properties of anion-disordered materials.

## 1 Introduction

Heteroanionic materials, which contain two or more anionic species, offer compositional and structural flexibility not found in otherwise analogous homoanionic materials.^1−4^ As such, controlling the relative stoichiometries and crystallographic arrangements of the anion species in heteroanionic materials allows their properties to be tuned.^5,6^ This compositional and structural versatility means that heteroanionic materials find applications across a range of critical technologies, including thermoelectrics,^7^ photocatalysis,^8,9^ and energy storage.^10−13^

The properties of heteroanionic materials depend on their chemical composition, specifically the identities and relative stoichiometries of their constituent anions, and on their structure, particularly the arrangement of these anions within their host crystal structure. While some heteroanionic materials are crystallographically ordered, with their constituent anion species arranged in a regular, repeating pattern, others are crystallographically disordered, with their anion species randomly distributed across crystallographically equivalent sites. In these anion-disordered systems, at long range, the site occupations of these anion species are uncorrelated. At short range, however, these different anion species often exhibit short-range ordering, characterized by one or more local configurations of anions appearing more frequently than in a fully uncorrelated (maximum entropy) anion distribution.

While experimental techniques that probe long-range correlations between atoms, such as X-ray or neutron Bragg scattering, can determine the average crystal structure of anion-disordered materials, these methods cannot resolve any short-range ordering, if present. Instead, these methods yield only an effective unit cell where each anion site is occupied by a statistical average of the constituent anion species. Short-range structural information can be obtained from scattering experiments in the form of pair distribution function (PDF) data.^14^ However, for heteroanionic materials containing anions with similar X-ray or neutron scattering factors, such as oxyfluorides, it is often not possible to assign anion site occupations based solely on PDF data, and alternative methods must be used to resolve the anionic structure of these materials.

One method that has proven effective for studying the short-range structure of heteroanionic materials is solid-state nuclear magnetic resonance (NMR) spectroscopy, which provides direct information about the local chemical environments of individual chemical species. In the case of oxyfluorides, the use of NMR spectroscopy is facilitated by the high gyromagnetic ratio and broad chemical shift range of the sole natural isotope of fluorine, ^19^F, and several previous studies have used ^19^F NMR spectroscopy to study oxygen–fluorine ordering in oxyfluorides.^15−27^ However, using ^19^F NMR data alone to unambiguously determine O/F ordering in disordered oxyfluorides can be challenging, due to the large number of possible anion permutations that might need to be considered; as a consequence, complementary experimental or computational data are often required to fully solve the anion structure.

Another approach to probing short-range order in heteroanionic materials is to use computational electronic structure methods, such as Density Functional Theory (DFT).^28,29^ By calculating the relative energies of structures with varied anion configurations, low-energy anion structures can be identified directly. The high computational cost of electronic structure methods, however, limits their use to relatively small computational cells and to relatively small numbers of possible anion orderings, making it difficult to fully characterize the anion substructure in partially disordered materials. In these cases, it is necessary to use alternative computational methods that accurately describe correlations in anion site occupations at length scales beyond those typical of electronic structure calculations and ideally allow for rapid evaluation of possible anion arrangements.

**Figure 1:**
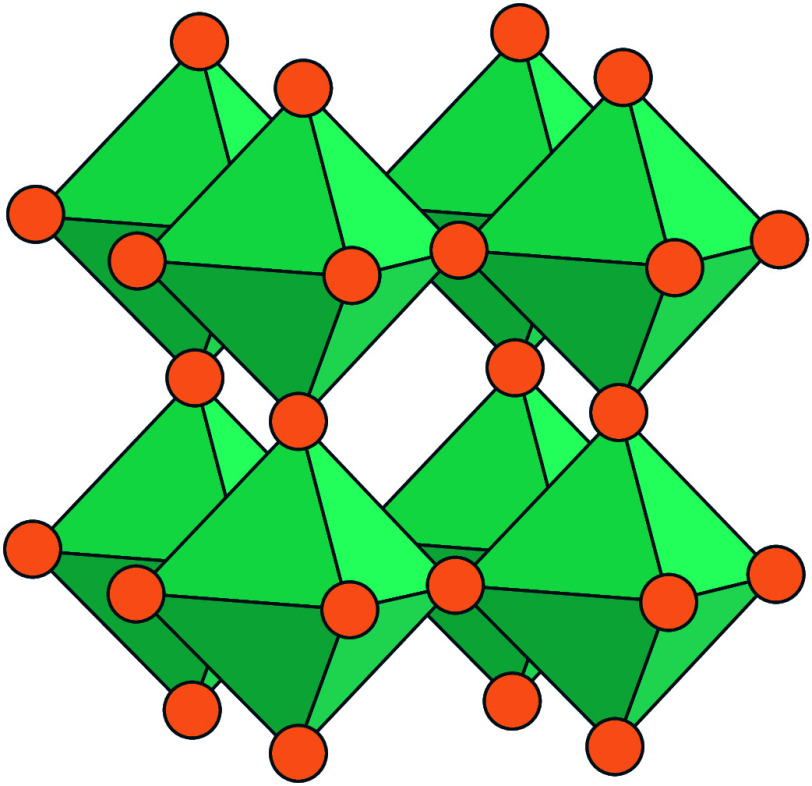
The cubic ReO_3_-type structure (space group *Pm*3̅*m*), comprised of corner-sharing MX_6_ octahedra.

Here, we report an investigation of the anionic structure in the anion-disordered transition-metal oxyfluoride, cubic (ReO_3_-type) TiOF_2_. Cubic TiOF_2_ has previously been studied as a lithium-ion electrode material^30,31^ and as a photocatalyst.^32,33^ The average structure of cubic TiOF_2_ consists of corner-sharing Ti[O,F]_6_ octahedra within a cubic *Pm*3̅*m* space group (Figure 1). A previous X-ray diffraction study of cubic TiOF_2_ found no evidence for anion ordering, and, on this basis, it was suggested that oxygen and fluorine are fully disordered (uncorrelated) over the available Wyckoff 3d sites.^34^ Short-range ordering of oxygen and fluorine anions, however, is known in other ReO_3_-type transition-metal oxyfluorides, such as NbO_2_F and TaO_2_F,^26,35−38^ and it is therefore reasonable to ask whether ReO_3_-type TiOF_2_ might also exhibit short-range anion ordering.

Using a combination of DFT calculations and cluster expansion modeling, we predict strong short-range ordering in ReO_3_-type TiOF_2_, characterized by an absence of collinear O–Ti–O units and a preference for polar *cis*-TiO_2_F_4_ titanium coordination. This polar coordination around titanium allows shorter Ti–O bonds and longer Ti–F bonds relative to the conventional *Pm*3̅*m* structure, which gives increased net bonding relative to *trans*-TiO_2_F_4_ titanium coordination. The preferential *cis*-TiO_2_F_4_ coordination also results in correlated anion disorder,^12,39,40^ which gives uncorrelated anion site occupations at longer distances, in agreement with the average *Pm*3̅*m* structure assigned from long-length-scale diffraction data.^34^

To validate our computationally predicted structural model, we use a genetic algorithm (GA) to generate structures with partial thermal disorder, which we use as structural models for as-synthesized ReO_3_-type TiOF_2_. We then compute simulated PDF and ^19^F NMR data for these GA-predicted structures and compare these to corresponding experimental PDF and ^19^F NMR data. To generate our simulated ^19^F NMR spectra, we convert from DFT-calculated magnetic shieldings to (calculated) chemical shifts using an empirical transformation function that we derive by fitting calculated magnetic shielding data for TiF_4_ to previously reported experimental ^19^F NMR data.^41^ For both the PDF and ^19^F NMR data, we observe good agreement between our simulated data for the GA-predicted structural model and our experimental data, supporting our computationally predicted structural model.

We also perform additional DFT calculations to evaluate how the degree of oxygen/fluorine ordering in ReO_3_-type TiOF_2_ affects its lithium intercalation properties. We find that increasing anion disorder makes lithium intercalation more favorable by, on average, up to 2 eV. This result suggests that the electrochemical properties of ReO_3_-type TiOF_2_, and potentially other heteroanionic intercalation electrode materials, can be controlled through synthesis protocols designed to produce samples with specific degrees of short-range anion order.

## 2 Methods

### 2.1 Experimental Details

TiOF_2_ was synthesized following the method described in ref (42). The synthesized compound was subsequently treated at 170 °C under vacuum to remove OH groups.

X-ray powder diffraction analysis was performed using a Rigaku Ultima IV X-ray diffractometer equipped with a Cu Kα radiation source (λ = 1.54059 Å). X-ray total scattering data were collected at the 11-ID-B beamline at the Advanced Photon Source, Argonne National Laboratory, using high-energy X-rays (λ = 0.2128 Å) up to a high momentum transfer value, *Q*_max_ = 18 Å^–1^.^43,44^ The raw total scattering data were processed using Fit2D.^45^ Pair distribution function (PDF) data, *G*(*r*), were derived by Fourier transformation after eliminating Kapton and background contributions using PDFgetX2.^46^ Refinement of the PDF data was performed using PDFgui,^47^ setting the *Q*_damp_ parameter at 0.04. The refined parameters included the lattice parameter, the scale factor, *s*_ratio_—the correction for the low-*r* to high-*r* PDF peak ratio due to correlated motion of bonded atoms^48^—and isotropic atomic displacement factors.

^19^F solid-state magic angle spinning (MAS) NMR experiments were performed on a Bruker Avance III spectrometer operating at 7.0 T (^19^F Larmor frequency of 282.2 MHz), using a 1.3 mm CP-MAS probe head. Room-temperature ^19^F MAS spectra were recorded using a Hahn echo sequence with an interpulse delay equal to one rotor period. The 90° pulse length was set to 1.55 μs, and the recycle delay was set to 20 s. ^19^F spectra were referenced to CFCl_3_ and fitted using the DMFit software.^49^

### 2.2 Computational Details

To model the relative energies of competing O/F anion configurations within the ReO_3_-type TiOF_2_ structure, we fitted a cluster expansion model to DFT-calculated energies of 65 symmetry-inequivalent 2×2×2 supercells. These 65 supercells were sampled from the complete set of 2664 symmetry-inequivalent 2×2×2 supercells of ReO_3_-type TiOF_2_, which we enumerated using bsym.^50^ These DFT calculations were performed using the VASP code,^51,52^ with a plane-wave cutoff energy of 700 eV and a 4×4×4 Monkhorst–Pack *k*-point grid. The interactions between core and valence electrons were described using the projector augmented wave method,^53^ with cores configurations of [Mg] for Ti, [He] for O, and [He] for F; for Li, all electrons were treated as valence. These calculations used the revised Perdew–Burke–Ernzerhof generalized gradient approximation (GGA) functional (PBEsol),^54^ with a Dudarev +*U* correction applied to the Ti d states (GGA+*U*).^55,56^ A value of *U*_Ti,d_ = 4.2 eV was used, as for previous calculations on TiO_2_,^57,58^ Li-intercalated TiO_2_,^59−61^ and Ti-deficient hydroxyfluorinated anatase TiO_2_.^62−64^

For our cluster expansion model training set, we performed full geometry optimizations, allowing changes to the cell shape and volume as well as internal atomic coordinates. Each geometry optimization was deemed converged when all atomic forces were smaller than 0.01 eV Å^–1^. Our cluster expansion model was fitted using the MAPS component of the ATAT code,^65,66^ which produced a model with 10 nonzero ECIs and a cross-validation score of 0.013 eV per structure.^67^ Additional information about the cluster expansion fitting and resulting model are provided in the Supporting Information (SI).

To quantify bond strengths for the structures in our training set of 65 2×2×2 supercells, we calculated Ti–(O/F) integrated crystal-orbital Hamilton populations (iCOHPs) using the LOBSTER code,^68−70^ with Vaspfitpbe2015 basis functions used to map the VASP plane-wave basis set onto local orbitals.

To validate our structural model, we generated larger TiOF_2_ structures (4×4×4 supercells) using a genetic algorithm (GA) for structure prediction. Our GA used a combination of elitist and proportionate selection, with selection probabilities based on a Boltzmann fitness function and energies of competing configurations calculated using our DFT-derived cluster expansion model. Full details of this GA are provided in the SI.

Using our GA structure prediction protocol, we generated four TiOF_2_ 4×4×4 supercells for validation against our experimental PDF and ^19^F NMR data. For each supercell, we initially relaxed atomic positions and cell volume (fixed cell shape) in VASP, using the parameters described above and a 2×2×2 Monkhorst–Pack *k*-point grid. For input structures for ^19^F NMR spectra calculations, we then performed a full optimization (atomic positions, cell volume, and cell shape) using CP2K,^71^ using the PBE GGA exchange-correlation functional^72^ and the DFT-D3 dispersion-correction method of Grimme et al.,^73^ which corrects for the overestimation of bond-lengths and cell volumes found for typical PBE calculations.^74^ The CP2K calculations used Goedecker–Teter–Hutter (GTH) pseudopotentials^75^ and TZVP Gaussian basis sets (MOLOPT library), with a charge density plane-wave expansion energy cutoff of 720 Ry.

The ^19^F magnetic-shielding tensors for these geometry-optimized TiOF_2_ 4×4×4 supercells were calculated using the GIPAW approach^76,77^ within VASP,^51,78^ using the PBE GGA exchange-correlation functional^72^ with a 550 eV plane-wave cutoff and a 2×2×2 Monkhorst–Pack *k*-point grid. The simulated ^19^F MAS NMR spectra were constructed from the DFT-calculated ^19^F magnetic shielding data using the procedure described in ref (79). For each fluorine atom, a MAS NMR spectrum was simulated, given the relevant experimental values of spin rate (64 kHz) and the magnetic field (7 T). The full MAS NMR spectrum for a given structural model was then obtained by summing the spectra for all the constituent fluorine atoms. The simulated ^19^F NMR spectra were computed using the fpNMR package.^79^

To generate simulated ^19^F NMR spectra from DFT calculations, it is necessary to convert calculated isotropic magnetic shieldings, σ_iso_, and magnetic shielding anisotropies, σ_csa_, to isotropic chemical shifts, δ_iso_, and chemical shift anisotropies, δ_csa_, respectively.^80^ Suitable transformation functions were obtained by fitting linear models for σ_iso_→δ_iso_ and for σ_csa_→δ_csa_ using linear least-squares regression between calculated magnetic shielding data for TiF_4_ and corresponding chemical shift data previously reported.^41^ A full discussion of the derivation of these transformation functions is given in Section 3.4.4.

The calculation of the ^19^F magnetic shielding tensors for TiF_4_ was performed using the GIPAW approach^76,77^ within VASP.^51,78^ The TiF_4_ input structure for these reference magnetic shielding calculations was obtained from a geometry optimization performed in VASP,^51,78^ where only atomic coordinates were relaxed, keeping the cell parameters fixed to experimental values. This geometry optimization used Ti [Ne] and F [He] pseudopotentials and the Perdew–Burke–Ernzerhof (PBE) GGA functional, augmented with the DFT-D3 correction of Grimme et al.,^73^ to account for dispersion interactions between the isolated columns of corner-linked TiF_6_ octahedra that comprise the TiF_4_ structure.^41,81^ Both the geometry optimization calculation and the subsequent ^19^F NMR calculation used a plane-wave cutoff of 550 eV and a 1×6×3 Monkhorst–Pack *k*-point grid.

Additional results from calculations of the ^19^F magnetic shielding tensors for TiF_4_, performed with optimization of atomic positions but without the DFT-D3 correction, using CASTEP^82,83^ (to replicate the previous calculations of Murakami et al.^41^) and VASP, are provided in the SI. The SI includes additional details on the effects that different optimization and relaxation protocols have on the resulting TiF_4_ structure.

Lithium intercalation calculations were performed for three exemplar 4×4×4 TiOF_2_ supercells: one genetic-algorithm (GA)-predicted structure, a 4×4×4 supercell special quasi-random structure, and a 4×4×4 expansion of the DFT-predicted lowest-energy 2×2×2 structure. For each structure, we considered lithium intercalation at all nonequivalent cubic interstitial sites and performed geometry relaxations with lattice parameters fixed to those of the corresponding stoichiometric TiOF_2_ model. These calculations used a cutoff energy of 500 eV and a 2×2×2 Monkhorst–Pack *k*-point grid. To calculate lithium intercalation energies, elemental (metallic) lithium was modeled using a Li_2_ cell, with a cutoff energy of 500 eV and a 16×16×16 Monkhorst–Pack *k*-point grid.

## 3 Results and Discussion

### 3.1 X-ray Diffraction and PDF Analysis

Our X-ray diffraction data index to a *Pm*3̅*m* structure (SI Figure S1) with a cell parameter of *a*_0_ = (3.8076 ± 0.0001) Å, consistent with the value of *a*_0_ = (3.798 ± 0.005) Å reported by Vorres and Donohue.^34^ This result corresponds to a ReO_3_-type structural model, comprised of symmetric corner-sharing TiX_6_ octahedra with a single Ti–X nearest-neighbor distance of 1.899 Å.

Given the different formal charges of O^2–^ and F^–^, these anions are expected to exhibit differentiated bonding with Ti, resulting in distinct Ti–O and Ti–F bond lengths. In an anion-ordered system, differences in Ti–O and Ti–F bond lengths should theoretically be observable in the long-range diffraction data as a reduction in crystal symmetry from *Pm*3̅*m*. However, the absence of any observable deviation from *Pm*3̅*m* symmetry in our X-ray diffraction data indicates that, at long ranges, the positions of oxygen and fluorine are uncorrelated, giving an average high-symmetry *Pm*3̅*m* structure. This observation aligns with the previous study by Vorres and Donohue,^34^ wherein the absence of long-range O/F correlations in ReO_3_-type TiOF_2_ was interpreted as evidence that O and F are randomly distributed across the available Wyckoff 3d positions.

**Figure 2:**
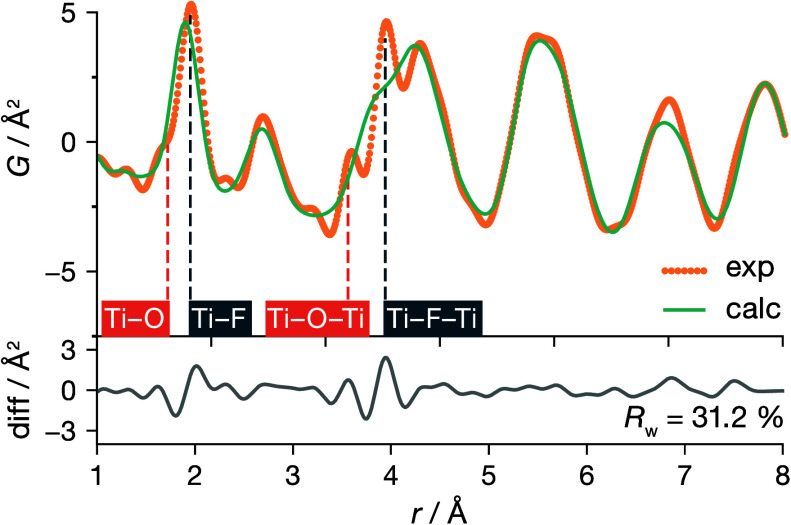
PDF refinement of cubic TiOF_2_ using the cubic ReO_3_-type (*Pm*3̅*m*) structure model.

To better understand the anionic substructure of ReO_3_-type TiOF_2_, we consider the pair distribution function obtained from X-ray total scattering data. For interatomic distances between 8 and 40 Å, the PDF data are well described by a cubic *Pm*3̅*m* model (*R*_w_ = 13.2% (SI Figure S2), in agreement with the X-ray diffraction analysis above. The experimental PDF for *r* < 8 Å, however, gives a poor fit (*R*_w_ = 31.2%) when modeled with a cubic ReO_3_-type (*Pm*3̅*m*) structure (Figure 2), indicating deviations from the average ReO_3_-type structure at short range. Notably, we observe apparent splittings in the nearest-neighbor Ti–X peak at ∼1.9 Å and in the next-nearest-neighbor peak at ∼3.8 Å, suggesting distinct bonding environments. Based on the expectation that Ti–O bonding will, in general, be stronger than Ti–F bonding,^35^ we preliminarily assign the peaks at 1.71 and 1.94 Å to Ti–O and Ti–F nearest-neighbor pairs, respectively, and the peaks at 3.55 and 3.93 Å to Ti–(O)–Ti and Ti–(F)–Ti next-nearest-neighbor cation pairs, respectively.

### 3.2 ^19^F NMR

**Figure 3:**
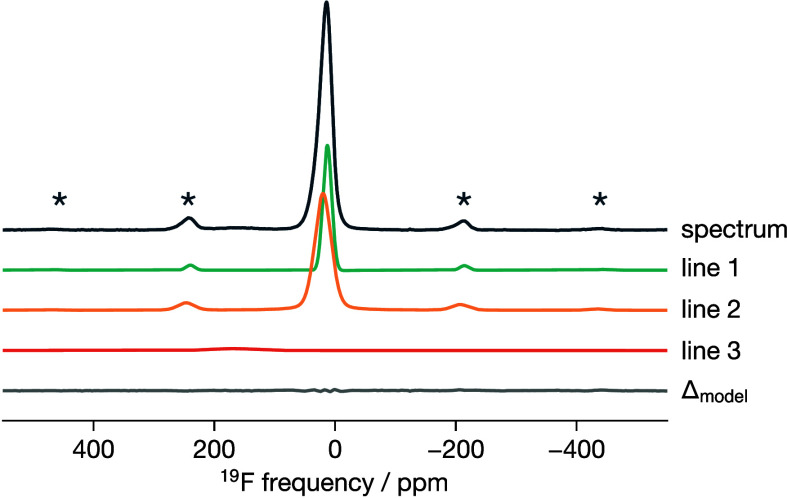
Experimental and fitted ^19^F MAS (64 kHz) NMR spectra of ReO_3_-type TiOF_2_. The individual resonances obtained from the fit are presented in Table I. Spinning sidebands of the main contribution are indicated by asterisks.

Figure 3 shows the ^19^F MAS solid-state NMR spectrum for ReO_3_-type TiOF_2_, which provides additional information about the local environments of the F^–^ anions. The spectrum shows a broad, slightly asymmetric main feature. We have reconstructed the experimental spectrum using two resonances (lines 1 and 2), which we assign to bridging Ti–F–Ti fluorine atoms. Additionally, our reconstruction reveals a broader and less intense line (line 3) at δ_iso_ ≈ 170 ppm, which we attribute to Ti–F–□ “non-bridging” fluorine atoms, where one adjacent titanium site is vacant.^42,84^

The asymmetry and width of the main peak in the ^19^F NMR spectrum suggest that our ReO_3_-type TiOF_2_ sample contains multiple distinct fluoride-ion environments. Previous studies have reported that TiOF_2_ prepared by aqueous solution synthesis contains hydroxyl defects and metal vacancies.^42^ The relative intensities of the fitted ^19^F NMR resonances (Table I) indicate that only 2.2% of F^–^ ions are “non-bridging” in our sample, implying a relatively high stoichiometric purity, with a Ti vacancy concentration of ≲0.5%.^85^ This low Ti-vacancy concentration is insufficient to explain the asymmetry and breadth of the main peak in the ^19^F NMR data. Instead, we interpret these features as indicative of O/F disorder, which is expected to produce a range of local fluoride-ion environments and a corresponding distribution of Ti–F bond lengths.

**Table I:** δ_iso_ (ppm), δ_csa_ (ppm), η_csa_, Line Widths (ppm), and Relative Intensities^a^

	δ_iso_	δ_csa_	η_csa_	LW	*I*	assignment
line 1	12.8	–159	0.65	17.1	32.0	Ti–F–Ti
line 2	20.0	–204	0.00	31.8	65.8	Ti–F–Ti
line 3	169.2	–113	0.00	80.6	2.2	Ti–F–□

a Averaging over lines 1 and 2 gives a weighted average for bridging Ti–F–Ti environments ⟨δ_iso_⟩ = 17.6 ppm.

### 3.3 DFT + Cluster Expansion Modeling

**Figure 4:**
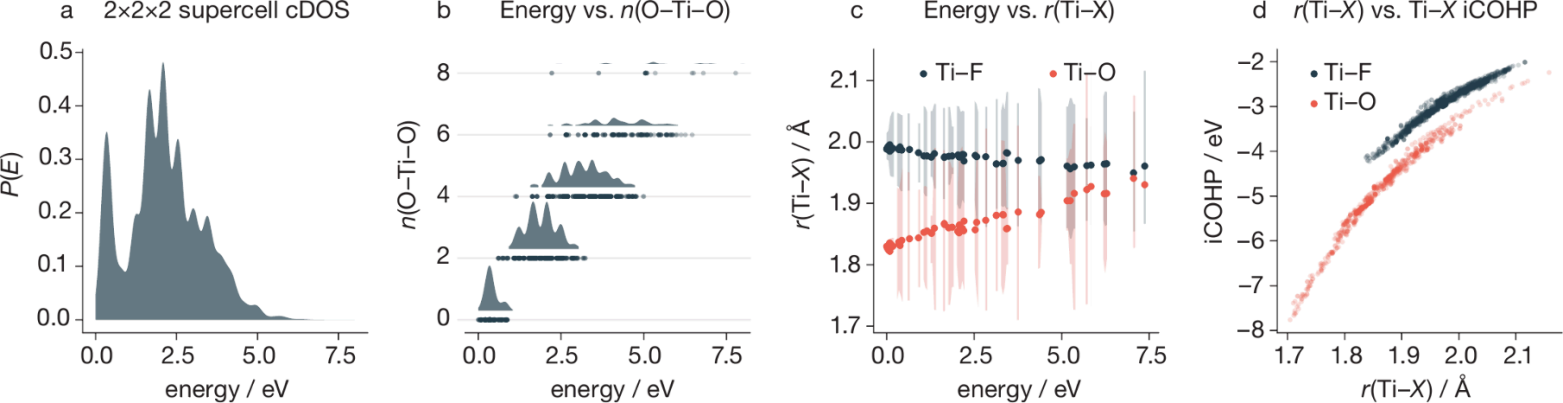
(a) Configurational density of states (cDOS) for a 2×2×2 supercell of TiOF_2_, calculated from our cluster expansion model. (b) Energies of 2×2×2 supercells of TiOF_2_, categorized by the number of collinear O–Ti–O units, and the corresponding cDOS contributions. (c) Ti–(O/F) bond lengths as a function of relative energy per structure for the set of 65 DFT-optimized 2×2×2 TiOF_2_ supercells. Solid circles show average Ti–O and Ti–F distances for each configuration. Vertical shading shows the 5th–95th percentile range for Ti–O and Ti–F distances, for all structures within a 0.1 eV moving window. (d) Bond length versus bond iCOHP for all Ti–O and Ti–F nearest neighbors in the set of DFT calculations. More negative iCOHP values correspond to stronger bonding.

To further explore the nature of O/F disorder in ReO_3_-type TiOF_2_, we conducted a computational analysis of all possible 2×2×2 TiOF_2_ supercells (Ti_8_O_8_F_16_), consisting of 2664 distinct symmetry-inequivalent O/F configurations. To efficiently compute the relative energies of all 2664 structures, we first performed DFT calculations on a subset of 65 structures and used these results to fit a cluster-expansion model, as described in the Methods section. This cluster-expansion model was then used to calculate the energies of all 2664 2×2×2 supercells. The resulting configurational density of states for all 2×2×2 TiOF_2_ supercells (Figure 4a) reveals an energy difference of 0.94 eV per formula unit between the configurations with the lowest and highest energies. This energy variation between different anion configurations indicates a strong energetic preference for certain short-range anion configurations over others, consistent with short-range ordering. This finding contradicts the previously proposed structural model that O and F positions in ReO_3_-type TiOF_2_ are completely uncorrelated,^34^ which instead implies equal energies for all 2×2×2 TiOF_2_ cells.

ReO_3_-type TiOF_2_ is chemically and structurally similar to ReO_3_-type NbO_2_F and TaO_2_F. In these materials, it has been proposed that collinear F–*M*–F units are disfavored and that oxygen and fluorine anions preferentially adopt short-range orderings that give asymmetric F–*M*–O units.^35^ This coordination asymmetry allows the central cation to shift off-center to form shorter Ti–O bonds, which has been suggested to increase the overall bonding strength of the *M*(O/F)_6_ unit. By analogy, we might anticipate that, in TiOF_2_, collinear O–Ti–O units are disfavored compared to asymmetric collinear O–Ti–F units. Figure 4b shows the distribution of energies for all 2×2×2 supercells, grouped by the number of collinear O–Ti–O units in each structure, out of a maximum of 8 possible for this supercell size. In general, structures with a greater number of collinear O–Ti–O units have higher configurational energies, while the lowest energy structures have no collinear O–Ti–O subunits. This observed correlation supports the hypothesis that, in ReO_3_-type TiOF_2_, oxygen and fluorine preferentially organize to give asymmetric collinear O–Ti–F units.

**Figure 5:**
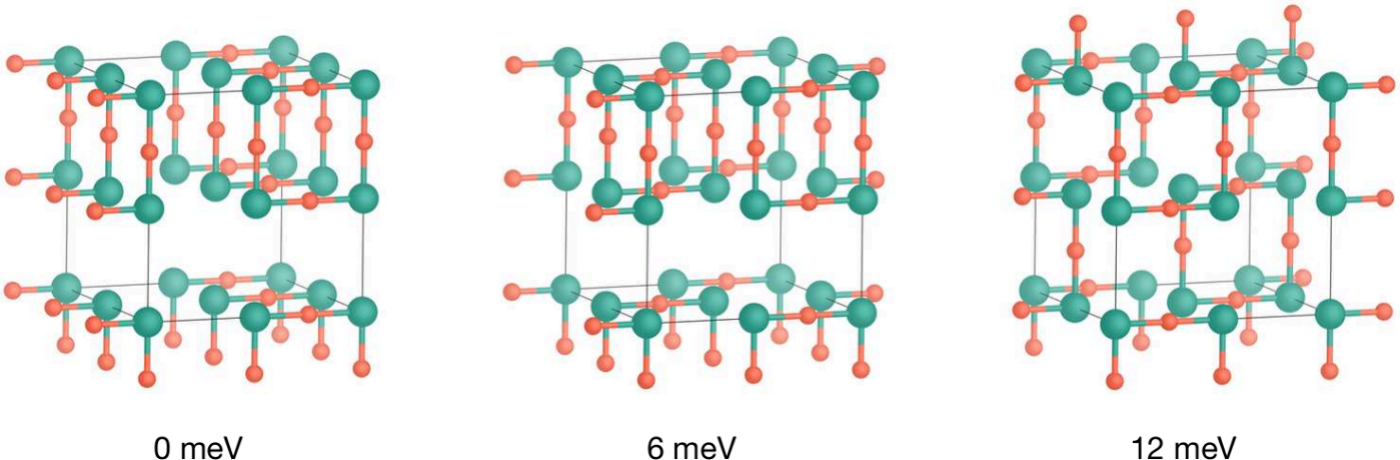
The three lowest-energy 2×2×2 supercells of TiOF_2_ predicted by the DFT-parametrized cluster expansion model and their relative energies per TiOF_2_ formula unit.

Figure 5 displays the three lowest energy 2×2×2 TiOF_2_ structures, all of which are comprised entirely of *cis*-Ti-[O_2_F_4_] subunits. This local coordination achieves local electroneutrality, in accordance with Pauling’s second rule,^86^ while also avoiding collinear O–Ti–O subunits. The energy difference between these three low-energy structures is only 12 meV, and all 2×2×2 structures containing only *cis*-Ti-[O_2_F_4_] units are within 80 meV of the lowest energy structure. Consequently, our calculations predict that ReO_3_-type TiOF_2_ exhibits a preference for polar *cis*-Ti-[O_2_F_4_] coordination, but these *cis*-Ti-[O_2_F_4_] units are expected to adopt a variety of different relative arrangements within the crystal structure, resulting in correlated anion disorder.^12,39,40^

The preference for polar *cis*-Ti-[O_2_F_4_] over nonpolar *trans*-Ti[O_2_F_4_] coordination in ReO_3_-type TiOF_2_, as predicted here, echoes the preference for polar cation coordination reported in other transition-metal oxyfluorides and oxynitrides.^1,29,35,87−95^ In these materials, the preference for *cis*- versus *trans*-MX_2_Y_4_ or *fac*- versus *mer*-MX_3_Y_3_ coordination has been attributed to the property of a polar configuration of anions: the central cation can move off-center, resulting in shorter M–O distances and stronger net cation–anion bonding. Our results for ReO_3_-type TiOF_2_ are consistent with this model—the lowest energy structures from our calculations, which are composed entirely of *cis*-Ti-[O_2_F_4_] units, have short Ti–O bonds (∼1.82 Å) and longer Ti–F bonds (∼1.99 Å), in agreement with our assignment of the split first peak in our experimental short-range PDF data (Figure 2). Further evidence for lower energy anion configurations being those that allow shorter Ti–O bonds and longer Ti–F bonds is provided by Figure 4c, which plots the Ti–O and Ti–F bond lengths for all 65 DFT-optimized 2×2×2 supercells in the cluster-expansion model training set as a function of energy relative to the lowest energy structure. In general, lower energy structures have shorter mean Ti–O bonds and slightly longer mean Ti–F bonds, whereas in the highest energy structures the mean Ti–O and mean Ti–F bond lengths are nearly identical (Figure 4c).

To further quantify the relationship between the Ti–(O/F) bond lengths and bonding strength, we calculated integrated iCOHPs for each Ti–(O/F) bond in our DFT data set, which are plotted against the corresponding bond lengths in Figure 4d. iCOHP values serve as indicators of bonding strength, with more negative values attributed to stronger and more covalent bonding.^68−70^ Both Ti–O and Ti–F bonds are predicted to become stronger as the Ti–(O/F) distance decreases. Moreover, both plots of bond strength versus bond length are concave, which indicates that Ti centers with a mix of shorter-than-average and longer-than-average bonds Ti–X bonds have greater net bond strength than Ti centers where all six Ti–X bonds are of equal length.

### 3.4 Genetic-Algorithm Structure Prediction, Intermediate-Range Ordering, and Validation against Experimental Data

#### 3.4.1 Genetic-Algorithm Structure Prediction

The DFT and cluster-expansion analysis detailed above (Section 3.3) predicts that ReO_3_-type TiOF_2_ exhibits short-range anion order characterized by preferential *cis*-Ti[O_2_F_4_] coordination. Our calculations also provide an explanation for this preference: anion configurations that avoid collinear O–Ti–O units allow local distortions from TiX_6_ coordination with six equal-length Ti–X bonds, resulting in shorter Ti–O (and consequently longer Ti–F) bonds, thereby increasing the net Ti–X bonding.

The energetic preference for *cis*-Ti[O_2_F_4_] short-range ordering implies a ground-state structure with 100% *cis*-Ti[O_2_F_4_] coordination, which is consistent with the lowest energy 2×2×2 structures shown in Figure 5, each of which exhibits 100% ordered *cis*-Ti[O_2_F_4_] units. The calculated configurational density of states (cDOS) (Figure 4a), however, does not show a clear energy gap above the lowest energy 2×2×2 structure, and we instead predict multiple low-energy structures that may be expected to be competitive under synthesis.^12^ Consequently, as-synthesized samples of ReO_3_-type TiOF_2_ are expected to exhibit some degree of partial disorder, while still demonstrating a general preference for local *cis*-Ti[O_2_F_4_] coordination.

**Figure 6:**
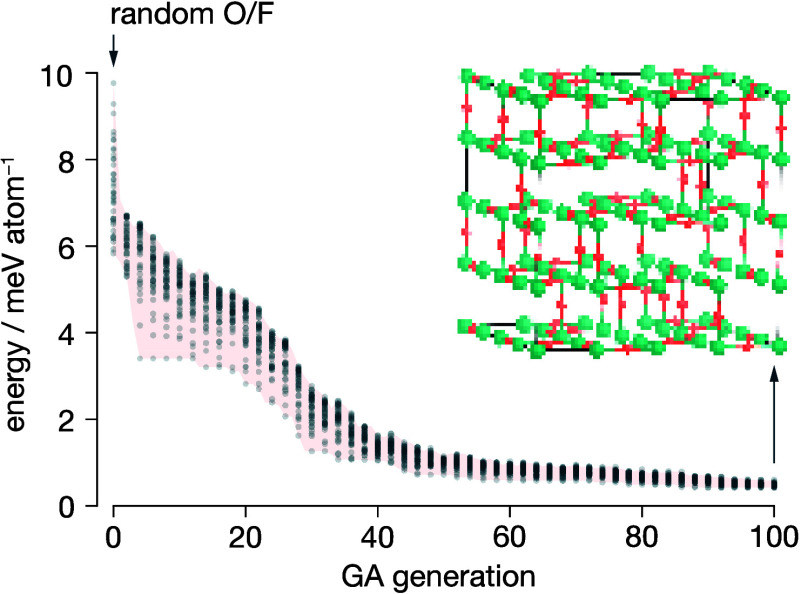
Evolution of energies of a population of 40 structures over 100 generations of the genetic algorithm structure prediction procedure. Generation 0 is initialized with a population of structures with random O/F configurations. Each shaded point shows the energy of a single structure within each generation. The inset shows one example supercell obtained as the lowest-energy structure after 100 GA generations.

To create structural models that incorporate this partial disorder, we used a genetic algorithm (GA) structure prediction scheme to generate a set of exemplar 4×4×4 supercells. For each structure prediction calculation, we initialized the GA with a starting population of 40 4×4×4 TiOF_2_ supercells, each with random O and F anion configurations. The GA used a combination of elitist selection and proportional selection, employing a Boltzmann-weighted fitness function, *f*_*i*_ ∝ exp(*E*_*i*_/*kT*), to select structures from each generation for seeding the next generation (full details of the GA algorithm are given in the SI). The energies for each structure considered by the algorithm were calculated using our DFT-derived cluster expansion model. This GA structure prediction scheme is conceptually similar to running multiple concurrent Monte Carlo-based simulated annealing simulations, where structural motifs associated with low-energy configurations at any point can be shared across simulations. The GA algorithm quickly filters out high-energy, less probable structures to produce a pool of structures with energetically reasonable O/F anion configurations (Figure 6).

Using this GA procedure, we generated four 4×4×4 supercells, with each selected as the lowest energy structure after 100 GA generations. The resulting 4×4×4 structures feature no collinear O–Ti–O units and predominantly exhibit *cis*-TiO_2_F_4_ coordination (93.0%), with some *fac*-TiO_3_F_3_ (3.5%) and TiOF_5_ (3.5%) coordination (SI Figure S6). Additional analysis of the TiX_6_ coordination geometries in these 4×4×4 models (see the SI) shows that the anions show small average deviations from ideal octahedra, while the mean Ti–O and Ti–F distances are significantly different, due to large off-center displacements of the Ti cations (averaging 0.20 Å).

#### 3.4.2 Intermediate-Range Anion Ordering

Like cubic TiOF_2_, NbO_2_F also adopts an average ReO_3_-type structure^26,96^ with oxygen and fluorine distributed over the Wyckoff 3d positions. NbO_2_F is believed to exhibit short-range ordering somewhat analogous to that predicted here for TiOF_2_, with collinear F–Ti–F units disfavored.^35,36^ Electron diffraction data for NbO_2_F, however, show ⟨*hk*1/3⟩* sheets of diffuse intensity, which has been attributed to anion ordering in one-dimensional strings along each of the three ⟨001⟩ directions.^36^ To explain this experimental observation, Brink et al. proposed a structural model for NbO_2_F in which oxygen and fluorine are ordered along ⟨001⟩ strings in repeating [F–O–O–F] sequences but are uncorrelated between pairs of ⟨001⟩ strings, regardless of whether these strings are aligned along the same or different ⟨001⟩ directions.^36^

Motivated by this evidence for [F–O–O–F] anion ordering along ⟨100⟩ strings in ReO_3_-structured NbO_2_F,^97^ we next considered whether ReO_3_-type TiOF_2_ can be predicted to exhibit analogous [O–F–F–O] ordering. To explore this possibility, we performed two sets of calculations. First, we used our GA structure prediction scheme with our DFT-derived cluster expansion model to generate a 6×6×6 supercell, with this supercell size chosen to accommodate anion orderings with a ×3 unit cell repeat distance. We then analyzed the resulting structure to determine the relative prevalence of different ⟨100⟩ orderings. Second, we computed DFT geometry-optimized energies for three sets of TiOF_2_ structures with different supercell sizes (2×2×2, 3×3×3, and 4×4×4) and different ⟨100⟩ anion orderings to determine whether any of these ⟨100⟩ anion orderings is sufficiently energetically favored to predict general anion ordering.

The first calculation, using our GA structure prediction scheme, yielded a 6×6×6 supercell with the same local coordination preferences as for the GA-predicted 4×4×4 supercells. The resulting structure contains no collinear O–Ti–O units, and the TiX_6_ coordination octahedra are predominantly *cis*-TiO_2_F_4_ (89.8%), with small proportions of *fac*-TiO_3_F_3_ (5.1%) and TiOF_5_ (5.1%). This distribution of TiO_*x*_F_6–*x*_ coordination octahedra differs significantly from that predicted by mapping the Brink et al.^36^ NbO_2_F model to TiOF_2_, with *cis*-TiO_2_F_4_ (44.4%), *fac*-TiO_3_F_3_ (29.6%), TiOF_5_ (22.2%), and TiF_6_ (3.7%) (SI Figure S8).

Furthermore, in our 6×6×6 GA-predicted model, only 25 out of 108 ⟨001⟩ columns (23.1%) exhibit [O–F–F–O] ordering, while the Brink et al.^36^ NbO_2_F-type model predicts that all ⟨001⟩ columns should exhibit this ordering. While our 6×6×6 GA-predicted structure disagrees with both the distribution of cation coordination environments and the distribution of anion orderings along ⟨100⟩ columns predicted by the NbO_2_F-type model, we do observe partial intermediate-range ordering. The proportion of columns with [O–F–F–O] anion sequences (23.1%) is higher than that expected for an equivalent supercell with a fully random arrangement of anions (6.6%). We attribute this effect to a second-order consequence of the short-range ordering in TiOF_2_, where collinear O–Ti–O units are strongly disfavored, resulting in (partial) anion correlations at intermediate length scales.

Although our GA-predicted 6×6×6 supercell suggests that TiOF_2_ does not exhibit NbO_2_F-type exclusive [O–F–F–O] ordering along ⟨100⟩ columns, this result might be a consequence of our choice of fitting procedure for the DFT-derived cluster expansion model used in the GA structure prediction scheme. Because our DFT training set includes only 2×2×2 supercells, only anion–anion interactions that fit within this supercell size are included in the resulting cluster expansion model.

To validate the predictions from our 6×6×6 GA structure prediction, we performed additional DFT calculations on a set of [O–F–F–O]-ordered 3×3×3 TiOF_2_ supercells and compared the resulting energies (per formula unit) to those of the 2×2×2 supercells in our CE training set with exclusive *cis*-TiO_2_F_4_ cation coordination and to the DFT-optimized energies of our four 4×4×4 GA-predicted supercells. All three sets of structures have no collinear O–Ti–O units and, therefore, are expected to all have relatively low energies, but they have different anion ⟨100⟩ orderings: The 2×2×2 all-*cis*-TiOF_2_ structures have exclusive [(F)–O–F–(O)] ⟨100⟩ ordering, the 3×3×3 structures have, by construction, exclusive [O–F–F–O] ordering, and the 4×4×4 GA-predicted supercells have a mixture of [O–F–F–F–O] and [O–F–O–F–O] ⟨100⟩ orderings.

By comparing the relative energies from all three sets of structures, we can directly test the hypothesis of favored Brink-type [O–F–F–O] anion-ordering in ReO_3_-type TiOF_2_. Under this hypothesis, we would expect the 3×3×3 [O–F–F–O] structures to have significantly lower energies than any of the 2×2×2 and 4×4×4 structures, which would predict that [O–F–F–O] ⟨100⟩ ordering forms preferentially during synthesis.

**Figure 7:**
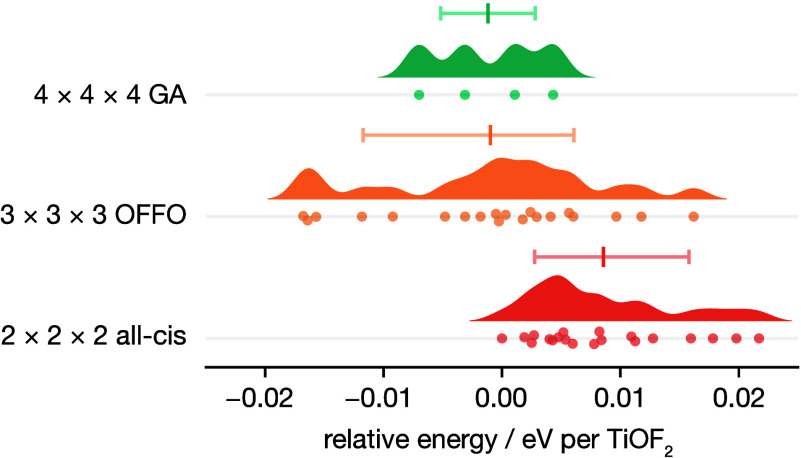
Raincloud plot^98^ showing DFT-calculated relative energies per formula unit of (top) the 4×4×4 GA-predicted structures; (middle) 20 randomly generated 3×3×3 structures with 100% [O–F–F–O] ordering along the ⟨100⟩ columns; (bottom) the 100% *cis*-TiO_2_F_4_ 2×2×2 supercells from the CE model DFT training set. Energies are given relative to the energy per formula unit of the lowest-energy 2×2×2 supercell. Error bars show the 16th–84th percentile range and mean relative energy per TiO_2_F_4_ unit for each data set.

Figure 7 shows our DFT-calculated energies per TiOF_2_ formula unit for each set of structures. While the lowest energy structures of those computed are 3×3×3 [O–F–F–O]-ordered structures, these are not significantly more stable than the 2×2×2 and 4×4×4 structures, which both have no [O–F–F–O] ordering. All computed structures are within 0.04 eV per formula unit of the lowest energy structure. Moreover, we find some 3×3×3 [O–F–F–O]-ordered structures with slightly higher energies per formula unit than some 2×2×2 and all 4×4×4 structures.

These DFT results are consistent with the predictions from our 6×6×6 GA structure prediction: we find no evidence for preferential [O–F–F–O] ordering along ⟨100⟩ columns in ReO_3_-type TiOF_2_. [O–F–F–O] ordering does yield low-energy structures by avoiding disfavored collinear O–Ti–O units, but it is predicted to be present within a mixture of ⟨100⟩ orderings, such as [O–F–O] and [O–F–F–F–O], that also satisfy this condition.

#### 3.4.3 GA Structure Validation versus PDF Data

To validate our computationally derived structural models for ReO_3_-type TiOF_2_, each GA-predicted 4×4×4 supercell was fully geometry-optimized using DFT and then used as input for direct comparison to our experimental PDF and NMR data.

**Figure 8:**
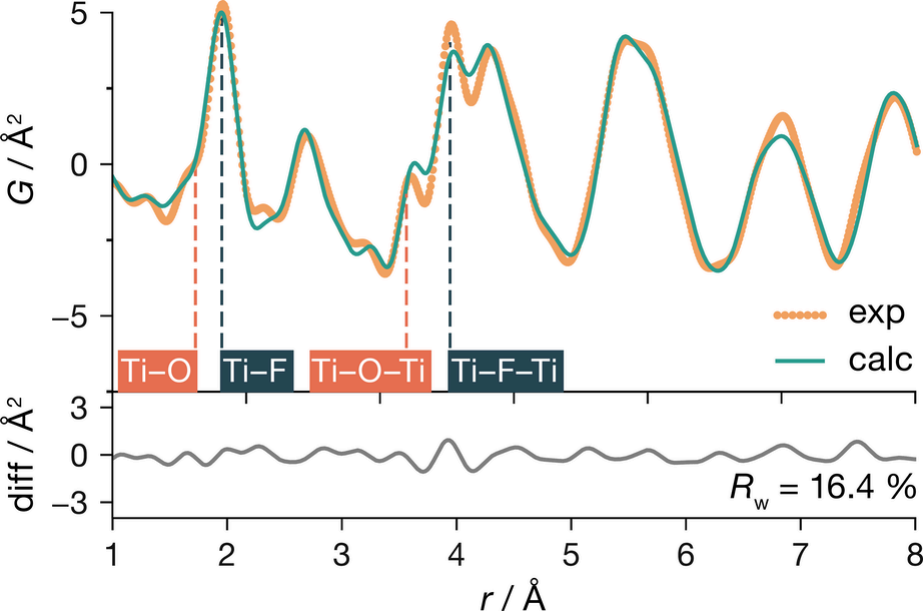
Comparison of the experimental PDF for cubic TiOF_2_ with a simulated PDF from a 4×4×4 supercell generated by our genetic-algorithm structure prediction (GA structure 4).

To validate against the experimental PDF data, each GA-predicted structure was used as an initial structural model that was fitted to the experimental PDF data, with the atomic positions left unrefined to limit the number of fitting parameters. All four GA-predicted structures give a better fit for the experimental data than the average cubic *Pm*3̅*m* model (*R*_w_ = 31.2%), with the best fit obtained for GA structure 4 (*R*_w_ = 16.4%) (Figure 8). The improved fit to the experimental PDF data is particularly evident in the split peak at 1.82 and 1.99 Å, which we previously assigned to nearest-neighbor Ti–O and Ti–F distances, respectively, and in the region between 3.5 and 4.0 Å, which we assigned to Ti–X–Ti pairs, and which is consistent with the observation from our 2×2×2 supercell DFT data set that ReO_3_-type TiOF_2_ preferentially adopts anion configurations that allow the Ti–O and Ti–F bond lengths to be shorter and longer, respectively, than the average Ti–X bond length of 1.90 Å. Our assignment of these peaks in the experimental PDF spectrum is also validated by direct analysis of the GA-predicted structures (SI), which gives mean Ti–O and Ti–F distances of 1.81 and 1.99 Å, respectively, and a clear splitting in Ti–X–Ti distances.

#### 3.4.4 GA Structure Validation versus ^19^F NMR MAS Data

To validate our GA-predicted structures against our experimental ^19^F NMR MAS data, we performed DFT calculations on each of the four GA-predicted structures to calculate magnetic shielding tensors for each fluoride anion. Obtaining a simulated ^19^F NMR spectrum from DFT calculations requires converting from calculated isotropic and anisotropic shielding values, σ_iso_ and σ_csa_, to predicted isotropic and anisotropic chemical shift values, δ_iso_ and δ_csa_, respectively.

For the isotropic values, the conventional approach to convert from σ_iso_ to δ_iso_ is to use a linear transformation function δ_iso_ = *a*σ_iso_ + *b*, where the parameters *a* and *b* are obtained by linear regression between computed σ_iso_ and experimental δ_iso_ data. Several different linear transformation functions for ^19^F have been published for a wide range of cations bonded to fluorine (see SI Table S4). One approach for the quantitative prediction of ^19^F δ_iso_ values for a disordered system containing one metal cation is to derive an appropriate linear transformation function from data for a reference ordered (oxy)fluoride containing the same cation as the system of interest and with fluoride ions occupying multiple crystallographic sites. This approach has previously been used to simulate the ^19^F NMR spectra of the disordered oxyfluorides MO_2_F^26^ and MOF_3_^95^ (M = Nb, Ta), with transformation functions derived by fitting to data for the corresponding MF_5_ fluorides.^74^

**Figure 9:**
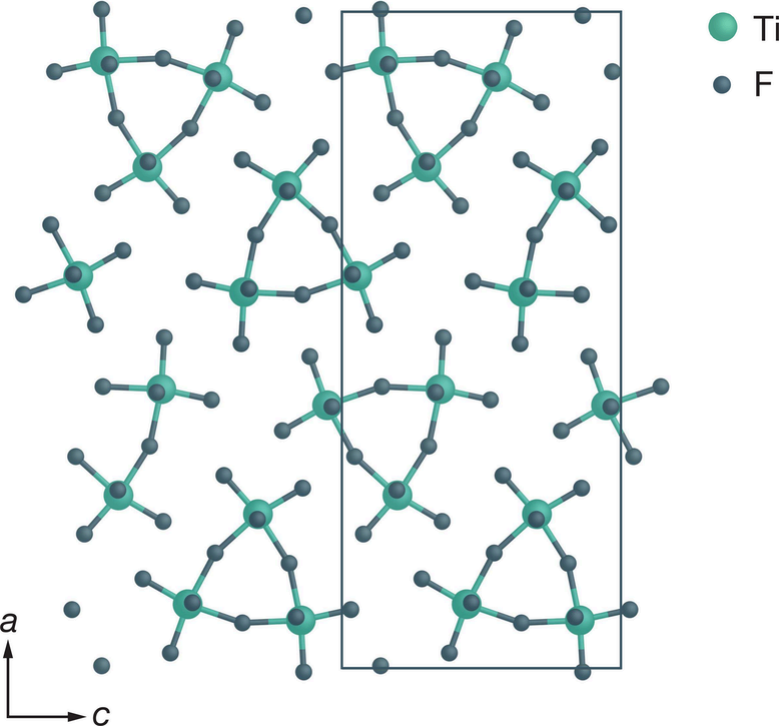
Structure of TiF_4_,^41,81^ showing the characteristic [Ti_3_F_15_] rings which are connected along the *b* axis, and containing distinct bridging Ti–F–Ti and terminal Ti–F fluorine environments. Adapted with permission from ref (41). Copyright 2019 Elsevier.

For obtaining a transformation function σ_iso_→δ_iso_ for titanium (oxy)fluorides, a reasonable reference system is TiF_4_. TiF_4_ is formed from corner-sharing TiF_6_ octahedra (Figure 9) and contains 12 inequivalent fluorine sites that can be classified into two groups: bridging fluorine atoms, which are bonded to two titanium centers, and terminal fluorine atoms, which are bonded to only one titanium center.^41,81^ High-resolution ^19^F NMR data for TiF_4_ have previously been reported by Murakami et al.,^41^ who derived a linear σ_iso_→δ_iso_ transformation function by fitting this model function to DFT-calculated σ_iso_ and experimental δ_iso_ data.^99^ While the DFT and experimental σ_iso_ and δ_iso_ data of Murakami et al. follow an approximately linear relationship, close inspection of these data (reproduced in SI Figure S10) shows that a single linear relationship is not able to accurately describe the correlations between σ_iso_ and δ_iso_ simultaneously for both bridging and terminal fluorine atoms, with each subset of atoms showing systematic deviations from the linear model obtained from fitting to all the fluorine atoms. Murakami et al. were also unable to obtain a satisfactory quantitative relationship between their calculated and experimental data for the magnetic anisotropies, which prevents the quantitative prediction of spinning side bands.

**Figure 10:**
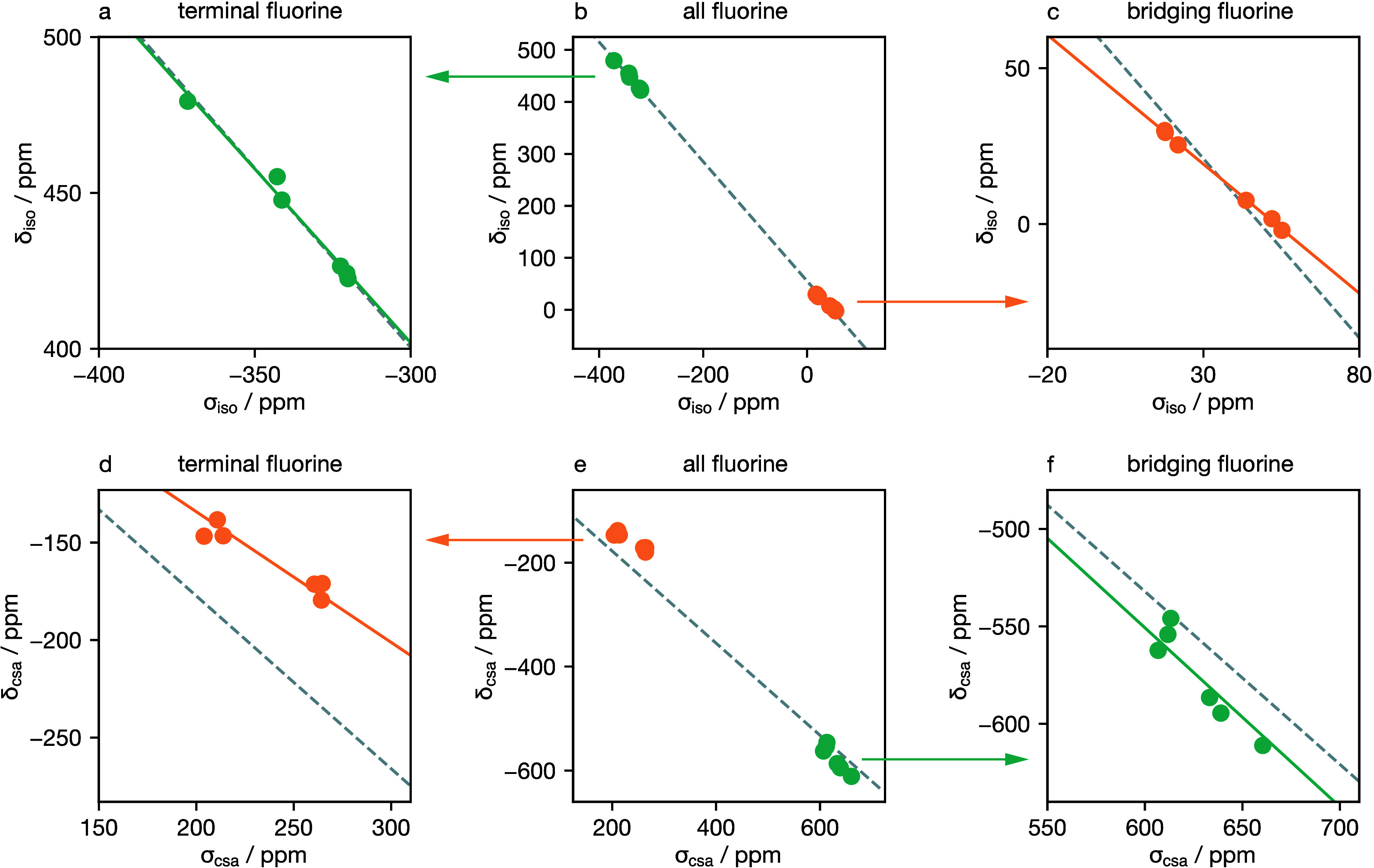
^19^F σ_iso_ and σ_csa_ values for TiF_4_ from DFT calculations (VASP, PBE + DFT-D3), plotted against the corresponding experimental δ_iso_ and δ_csa_ values, respectively.^41^ Panels (b) and (e) show all data and corresponding linear-least-squares fits (dashed lines). Panels (a) and (c), and (d) and (f), show the same data, selecting values for terminal F and bridging F only, respectively. Each panel shows the original linear model obtained from fitting to the full data set (dashed lines) and a revised linear model obtained by fitting to the corresponding data subset only (solid lines). The resulting best-fit linear models are δ_iso_ = −0.830σ_iso_ + 44.1 and δ_csa_ = −0.671σ_csa_ for bridging F, and δ_iso_ = −1.116σ_iso_ + 67.2 and δ_csa_ = −0.918σ_csa_ for terminal F.

To address these limitations, and to derive transformation functions suitable for the simulation of ^19^F NMR spectra of titanium (oxy)fluorides, we have revisited the analysis by Murakami et al.^41^Figure 10b shows calculated σ_iso_ data for TiF_4_ plotted against the corresponding experimental δ_iso_ values reported by Murakami et al.^41^ The data form two distinct clusters, corresponding to terminal and bridging F, at low and high σ_iso_ values, respectively. As reported by Murakami et al., the full data set does show an approximately linear relationship between σ_iso_ and δ_iso_ values. Zooming-in on the data for terminal and bridging fluorine atoms (Figure 10a and c, respectively), however, highlights the deficiencies of fitting a single linear model to both groups of data. The observation that these ^19^F NMR data are not well described by a single linear relationship is, perhaps, unsurprising, given the significant difference in local chemical environment and bonding for fluorine atoms directly bonded to two versus one Ti centers. To account for the categorical difference between bridging and nonbridging fluorine atoms, we fit separate linear models to the two clusters of data. This approach is much better able to quantitatively describe the correlation between σ_iso_ and δ_iso_ within each category of fluorine atoms (bridging versus nonbridging), and we obtain best-fit linear relationships of δ_iso_ = −0.830σ_iso_ + 44.1 for bridging F and δ_iso_ = −1.116σ_iso_ + 67.2 for terminal F.

Figure 10d–f shows an equivalent treatment of the DFT-calculated σ_csa_ and experimentally derived δ_csa_ data. Fitting the linear relationship δ_csa_ = *a*σ_csa_ to the full σ_csa_ versus δ_csa_ data set (Figure 10e) gives a poor fit, with the data for terminal and bridging fluorine atoms showing systematic deviations from the average best-fit model. By fitting separate functions to the data for the terminal (Figure 10d) fluorine atoms and for the bridging (Figure 10f) fluorine atoms, respectively, however, we obtain two transformation functions that much more accurately describe the quantitative relationship between σ_csa_ and δ_csa_ in TiF_4_.

In our GA-predicted models, all F’s bridge between two Ti’s. To generate simulated ^19^F NMR spectra for these GA-predicted TiOF_2_ structural models, we use the σ_iso_→δ_iso_ and σ_csa_→δ_csa_ relationships derived for bridging F in TiF_4_. We note that the σ_iso_→δ_iso_ and σ_csa_→δ_csa_ relationships derived here for terminal F species in TiF_4_ provide suitable transformation functions for nonbridging Ti–F–□ fluorines that may be present in other titanium fluorides and oxyfluorides.

**Figure 11:**
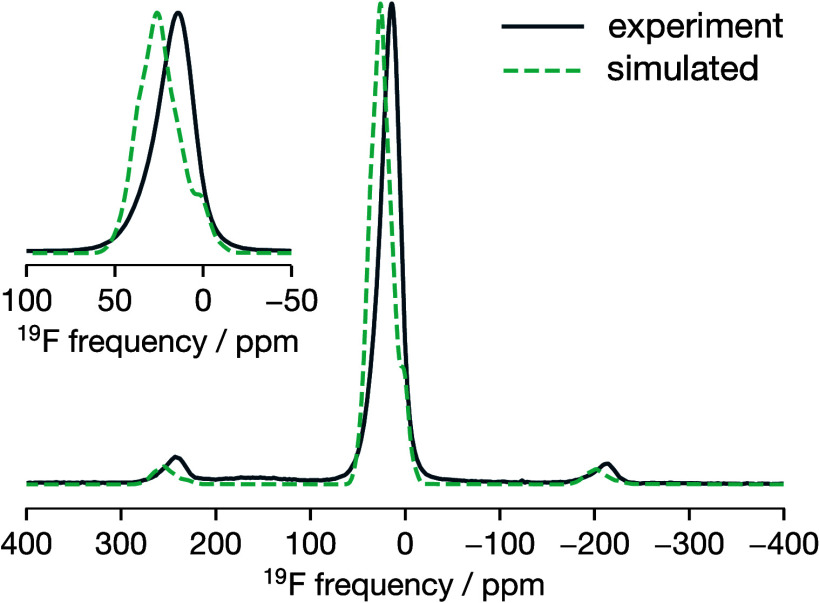
Experimental (solid line) and simulated (dashed line) ^19^F MAS (64 kHz) NMR spectra of ReO_3_-type TiOF_2_ (GA-predicted structure 4).

For all four GA-predicted structures, we obtain simulated ^19^F spectra that are in close agreement with the experimental TiOF_2_ spectrum (see Figure 11 for structure 4 and SI Figure S13 for all four GA-predicted structures), with average chemical shift values ranging from 22.6 to 25.4 ppm (SI Table S11). Moreover, the chemical shift anisotropies are well modeled, as evidenced by the correct reproduction of the spinning sidebands. The simulated and experimental spectra are not perfectly superimposed due to slightly different averages and larger spreads in the calculated chemical shift values compared to the experimental data. This discrepancy is not altogether unexpected, given the large chemical shift range of ^19^F (over 1000 ppm^100^), the inherent uncertainty in our σ_iso_→δ_iso_ relationship, and the use of finite-size 4×4×4 structural models as approximations to the experimental structure.

Both the simulated PDF and ^19^F NMR spectra show good agreement with the corresponding experimental data, with particularly good agreement in the case of the PDF data for GA-predicted structure 4. We therefore conclude that our DFT-derived cluster expansion model correctly describes the form of anion short-range order in ReO_3_-type TiOF_2_, and we consider structure 4, obtained from our GA structure prediction, as a representative structural model for the experimental samples considered in this work.

### 3.5 Lithium Intercalation and the Effect of O/F Order versus Disorder

ReO_3_-type TiOF_2_ has previously been considered as a potential lithium-ion electrode material.^30,31^ Our results above indicate that ReO_3_-type TiOF_2_ exhibits a specific short-range anion ordering consisting of preferential *cis*-TiO_2_F_4_ titanium coordination, which gives rise to correlated disorder at longer length scales. Our analysis of the relative energies of different anion configurations shows that there are a large number of low-energy anion configurations that are expected to be competitive under synthesis conditions (Figure 4), indicating that the precise anion configuration in ReO_3_-type TiOF_2_ might depend on the choice of synthesis protocols, suggesting a possible route to modulating technologically relevant properties, such as lithium intercalation behavior.

Having predicted and validated structural models for “as-synthesized” ReO_3_-type TiOF_2_, we now consider the effect of variation in local anion structure on lithium intercalation properties. To this end, we computed the dilute limit intercalation voltage for all possible interstitial sites in three exemplar structures with varying degrees of anion ordering: these structures comprise a 4×4×4 supercell of the lowest energy 2×2×2 structure, which is fully ordered with all-*cis*-Ti[O_2_F_4_] coordination, the partially disordered GA-predicted structure 4, and a maximally disordered 4×4×4 special quasirandom structure (SQS), which approximates the O/F correlations for an infinite lattice with a fully random (maximum-entropy) distribution of anions.^101,102^

**Figure 12:**
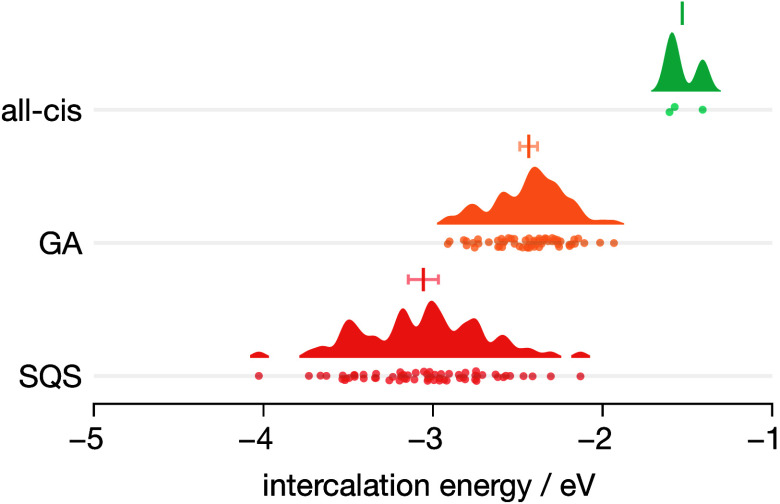
Effect of changing O/F substructure on lithium intercalation energies into cubic TiOF_2_. Data are shown as raincloud plots^98^ for three exemplar structures: (top) the fully ordered all-*cis*Ti[O_2_F_4_] structure; (middle) a 4×4×4 supercell genetic-algorithm-predicted structure (GA structure 4); and (bottom) the 4×4×4-supercell special quasirandom structure. For each data set, the points show individual calculated intercalation energies, and the solid distribution shows a kernel density estimate of the distribution of intercalation energies. Error bars show the 95% compatibility interval for the estimated mean of each data set, obtained by bootstrap resampling of the original data.^103^

Figure 12 presents calculated lithium intercalation energies and estimated mean values for each exemplar structure. The fully ordered all-*cis*-Ti[O_2_F_4_] structure has only three nonequivalent interstitial sites and therefore has a relatively narrow distribution of lithium intercalation energies, with a mean of −1.53 eV. GA-predicted structure 4 is partially disordered, and all 64 cubic interstitial sites in the 4×4×4 supercell are therefore inequivalent by symmetry. This gives a broader spread in lithium intercalation energies of more than ∼1 eV, with an estimated mean of (−2.44 ± 0.05) eV. The SQS structure is, again, more disordered than the GA-predicted structure and has an even broader spread in lithium intercalation energies (∼2 eV) and an estimated mean of (−3.06 ± 0.09) eV.

These results show that the lithium intercalation energy for ReO_3_-type TiOF_2_ is sensitive to the precise arrangement of oxygen and fluorine atoms within the host structure, with the mean intercalation energy shifting by >1.5 eV between the fully ordered all-*cis*Ti[O_2_F_4_] structure and the 4×4×4 special quasirandom structure considered here. In general, as the anion substructure becomes more configurationally disordered, the intercalation energy shifts to more negative values, while the distribution of lithium intercalation energies becomes broader. This result suggests that the electrochemical properties of ReO_3_-type TiOF_2_, and, by analogy, other heteroanionic intercalation electrode materials may be modulated through directed synthesis protocols that produce samples with different degrees of short-range anion order.

## 4 Summary and Conclusions

Heteroanionic materials offer a rich chemical space for developing new materials with targeted properties. To understand and control the properties of heteroanionic materials requires a detailed characterization of their structures—in particular, the specific arrangement of the component anions. Resolving the anionic substructure of anion-disordered oxyfluorides is particularly challenging because X-ray and neutron Bragg scattering experiments give only an average structural description. Resolving local structural details in anion-disordered oxyfluorides, therefore, requires using alternative complementary experimental or computational techniques.

Here, we have presented a study of the anionic substructure in the exemplar transition-metal oxyfluoride ReO_3_-type TiOF_2_, using a combination of X-ray PDF, ^19^F NMR, DFT modeling, and genetic-algorithm structure prediction. We find that ReO_3_-type TiOF_2_ exhibits strong short-range ordering characterized by preferential *cis*-O_2_F_4_ coordination around titanium. This *cis*-coordination of titanium allows titanium cations to move away from the center of their coordination octahedra to give shorter Ti–O bonds (and longer Ti–F bonds), giving a net increase in total Ti–X bond strength, relative to more symmetric *trans*-O_2_F_4_ titanium coordination. This preferential *cis*-TiO_2_F_4_ coordination also gives rise to correlated anion disorder,^39^ where the configuration of oxygen and fluorine ions decorrelates with separation, resulting in long-range anion disorder that is consistent with the average *Pm*3̅*m* structure model previously proposed from X-ray powder diffraction data.^34^

To obtain structural models that incorporate this correlated disorder, we used genetic algorithm structure prediction to generate partially disordered supercells. We then validated these structural models by generating simulated X-ray PDF and ^19^F NMR data, which we compared to equivalent experimental data for our synthesized TiOF_2_ sample. For the simulation of the ^19^F NMR spectrum, we used new empirical linear transformation functions to convert from calculated shielding values, σ_iso_ and σ_csa_, to predicted chemical shift values, δ_iso_ and δ_csa_, which we derived by fitting calculated σ_iso_ and σ_csa_ values for bridging fluoride ions in TiF_4_ to previously published experimental data.^41^ We expect the resulting linear transformation functions to be generally applicable for calculating ^19^F NMR spectra of other titanium oxyfluorides. For both the X-ray PDF and ^19^F NMR data, our simulated data agree well with the corresponding experimental data, indicating that our genetic-algorithm-predicted structures reproduce well the short-range structure of our sample.

We then considered the effect of variations in anionic short-range order on the lithium intercalation properties of ReO_3_-type TiOF_2_. By performing additional DFT calculations, we showed that the local anion substructure can have a significant effect on lithium intercalation voltages, with an example fully ordered low-energy structure and the maximally disordered special quasirandom 4×4×4 supercell structure showing a difference of mean lithium intercalation voltage of >1.5 V as well as a large increase in the spread of intercalation voltage values. Because the precise short-range structure of ReO_3_-type TiOF_2_ may be affected by different synthesis protocols, this result indicates that it may be possible to tune the electrochemical intercalation behavior of TiOF_2_—and, by analogy, of other transition-metal heteroanionic materials—through careful design of synthesis routes.

The work presented here demonstrates how the detailed local structure of heteroanionic oxyfluorides can be resolved using a combination of experimental and computational methods. By combining X-ray PDF analysis and ^19^F NMR spectroscopy with DFT modeling and GA structure prediction, we have identified a revised structural model for ReO_3_-type TiOF_2_ that is consistent between our experimental and computational analyses. We have also identified how the details of local coordination geometry and bonding direct short-range order in this material, through anions adopting local configurations that maximize Ti–(O/F) bond strength. The general strategy presented here is expected to be generally applicable to other anion-disordered oxyfluorides, where similar short-range deviations from the average crystallographic structure obtained from conventional diffraction methods are also likely.

## Data Availability

Data and plotting scripts for Figures 2–4, 6–8, and 10–12 are available on GitHub.^104^ This repository also includes CIF files for TiF_4_ optimized using DFT (atomic positions only), using CASTEP, VASP without DFT-D3, and VASP with DFT-D3, and inputs and outputs for all DFT calculations used to train the cluster expansion (CE) model, for the CE model training, for the genetic- algorithm structure prediction calculations, and for DFT calculations of lithium intercalation into the GA-predicted 4×4×4 TiOF_2_ supercell (model 4).

## Supporting Information Available

X-ray diffraction analysis for ReO_3_-type TiOF_2_; Pair Distribution Function data for TiOF_4_ between 8 and 40 Å; additional details of the cluster expansion model fitting and model ECIs; details of the genetic-algorithm structure prediction scheme; structural analysis of the GA-predicted structures; correlation between calculated σ_iso_ and experimental δ_iso_ values for ^19^F in titanium (oxy)fluorides; Haeberlen convention used to define the shielding and chemical shift NMR parameters; details about calculations using the NMR-CASTEP code; previously reported relationships between calculated σ_iso_ and experimental δ_iso_ values for ^19^F in inorganic fluorides; derivation of an empirical linear relation between calculated σ_iso_ and experimental δ_iso_ values for ^19^F in titanium (oxy)fluorides; effect of DFT calculation method on atomic positions for TiF_4_; and simulated ^19^F MAS NMR spectra for all four 4×4×4 GA-predicted structural models (PDF)
