# Supplementary material for: Correlated Anion Disorder in Heteroanionic Cubic TiOF2
Source: J Am Chem Soc. 2024 Jul 26;146(31):21889–902. doi: 10.1021/jacs.4c06304 (PMC11311215; doi:10.1021/jacs.4c06304)
Supplement: Supplementary file 1 — ja4c06304_si_001.pdf [file ja4c06304_si_001.pdf]

Supporting Information for “Correlated Anion-Disorder in Heteroanionic Cubic $TiOF_2$”

Christophe Legein,[1,*] Benjamin J. Morgan,[2,3,†] Alexander G. Squires,[4,3] Monique Body,[1] Wei Li,[5,6] Mario Burbano,[5,6] Mathieu Salanne,[5,6] Thibault Charpentier,[7] Olaf J. Borkiewicz,[8] and Damien Dambournet[5,6,‡]

[1] *Institut des Molécules et des Matériaux du Mans (IMMM), UMR 6283 CNRS, Le Mans Université, Avenue Olivier Messiaen, 72085 Le Mans Cedex 9, France*

[2] *Department of Chemistry, University of Bath, Claverton Down BA2 7AY, United Kingdom*

[3] *The Faraday Institution, Quad One, Harwell Science and Innovation Campus, Didcot OX11 0RA, United Kingdom*

[4] *Department of Chemistry, University College London, London, United Kingdom*

[5] *Sorbonne Université, CNRS, Physico-chimie des électrolytes et nano-systèmes interfaciaux, PHENIX, F-75005 Paris, France*

[6] *Réseau sur le Stockage Electrochimique de l'Energie (RS2E), FR CNRS 3459, 80039 Amiens Cedex, France*

[7] *Université Paris-Saclay, CEA, CNRS, NIMBE, 91191 Gif-sur-Yvette cedex, France*

[8] *X-ray Science Division, Advanced Photon Source, Argonne National Laboratory, Argonne, Illinois, USA*

(Dated: July 18, 2024)

CONTENTS

S1. X-ray diffraction analysis 2

S2. Pair distribution function 2
- A. PDF refinement 2
- B. Calculation of the reliability weighted R-Value, R_w 3

S3. Cluster expansion model: Additional details 3

S4. Genetic algorithm structure prediction 3

S5. Structural analysis of the GA-predicted $4 \times 4 \times 4$ supercell models 6

S6. Structural analysis of the GA-predicted $6 \times 6 \times 6$ supercell model 9

S7. Correlation between calculated σ_{iso} and experimental δ_{iso} values for ^{19}F in titanium (oxy)fluorides 11
- A. Haeberlen convention for the shielding and chemical shift NMR parameters 11
- B. Details about calculations using the NMR-CASTEP code 11
- C. Previously reported relationships between calculated σ_{iso} and experimental δ_{iso} values for ^{19}F in inorganic fluorides 11

S8. Derivation of an empirical linear relation between calculated σ_{iso} and experimental δ_{iso} values for ^{19}F in titanium (oxy)-fluorides 12

S9. Effect of DFT calculation method on atomic positions for TiF_4 13

S10. Additional ^{19}F NMR data for the $4 \times 4 \times 4$ GA-predicted structural models 14

Supporting Data 15

References 21

* christophe.legein@univ-lemans.fr
† b.j.morgan@bath.ac.uk
‡ damien.dambournet@sorbonne-universite.fr

S1. X-RAY DIFFRACTION ANALYSIS

Fig. S1 shows the Rietveld analysis of the X-ray diffraction pattern obtained for $TiOF_2$, using the ReO_3-type structure ($Pm\bar{3}m$).

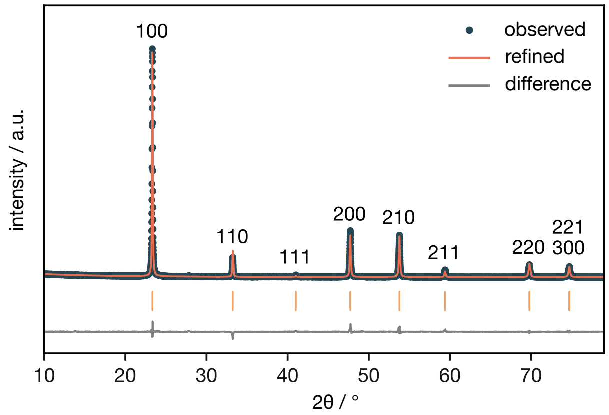

FIG. S1: Rietveld analysis of the X-ray diffraction pattern of $TiOF_2$ using the ReO_3-type structure (SG: $Pm\bar{3}m$). The grey curve shows difference between Rietveld-refined profile (orange line) and experimental X-ray diffraction pattern (black dots). The positions of Bragg reflections are shown by yellow vertical bars.

S2. PAIR DISTRIBUTION FUNCTION

A. PDF refinement

Fig. S2 shows PDF refinement of cubic $TiOF_2$ using the cubic ReO_3-type ($Pm\bar{3}m$) structure model from 8 Å to 40 Å.

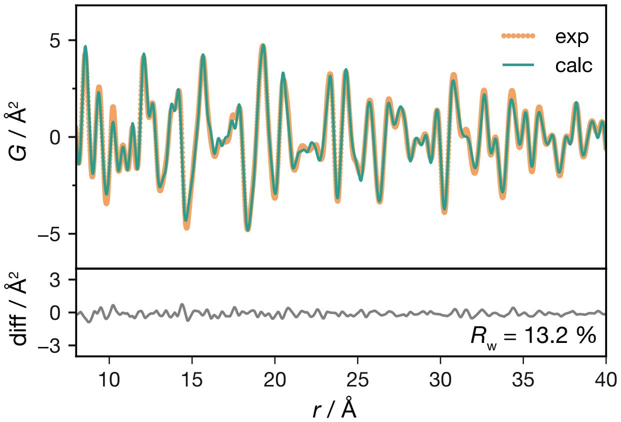

FIG. S2: PDF refinement using the cubic ReO_3-type model from 8 Å to 40 Å.

B. Calculation of the reliability weighted R-Value, R_w

The quality of a PDF refinement can be quantified by the reliability factor weighted R-value, R_w. The R-value describes the difference between the experimentally observed data and the fitted model data as a weighted average over data points. R_w is given by the R-value weighted for data point i:

$$R_\mathrm{w} = \sqrt{\frac{\sum_{i=1}^{N} w(r_i)[G_\mathrm{obs}(r_i) - G_\mathrm{calc}(r_i)]^2}{\sum_{i=1}^{N} w(r_i) G_\mathrm{obs}^2(r_i)}}, \tag{S1}$$

with G_obs and G_calc being the experimental and model PDF data, respectively, $w(r_i)$ the weight for each data point, and N the total number of data points.

S3. CLUSTER EXPANSION MODEL: ADDITIONAL DETAILS

We fit our cluster expansion (CE) model using an iterative structure selection procedure. We generated the complete set of 2664 symmetry inequivalent $2 \times 2 \times 2$ $TiOF_2$ supercell structures using BSYM [1], and initially selected 60 structures from this complete set, and computed their energies using DFT. We then fit an initial CE model and used this to predict the relative energies of the complete set of 2664 symmetry-inequivalent structures. Next, we identified the predicted highest and lowest energy structures. If these were not already in the DFT training set, we computed their energies using DFT and added them to the expanded training set before refitting the CE model. We repeated this procedure until our CE model did not predict new highest or lowest energy configurations not already in the CE training set. This procedure converged with a total of 65 structures. This approach of initial random sampling and iterative addition of predicted lowest and highest energy structures yields a training set that spans the full energy scale of candidate $2 \times 2 \times 2$ structures and includes structures with a broad range of local coordination geometries.

Fig. S3 shows a plot of DFT-calculated energies (relative to the lowest energy structure) versus corresponding CE-predicted relative energies for each structure in our CE training set. These structures span an energy range of $\sim 7.5\,\mathrm{eV}$, and our fitted cluster expansion model shows no systematic bias across this energy range. In Fig. S3 , we have shaded each point according to the number of *cis*-O_2F_4 coordination octahedra in each structure. This shading illustrates the general correlation between increasing the number of non-*cis*-O_2F_4 coordination environments and increased configurational energy (as discussed in the main manuscript) and visually demonstrates the range of coordination environments in our training set.

The cluster expansion model obtained from our fit has 10 non-zero ECIs, J_α. Fig. S4 plots the ECIs for all clusters of order > 1. For the five largest magnitude ECIs ($J_\alpha > |0.01|$ eV), the figure shows a schematic of one representative cluster. The local ordering predicted by any cluster expansion model results from the combined contributions of all the constituent ECIs, plus any implicit constraints: in this case, the stoichiometry is constrained to TiO_2F_4. It is notable, however, that the ECI with the largest magnitude corresponds to an effective interaction between X–Ti–Y collinear anion pairs. This ECI energetically penalises collinear X–Ti–X anion pairs where both anions are the same species (i.e., O–Ti–O or F–Ti–F) versus collinear anion pairs with mixed anion species (O–Ti–F).

S4. GENETIC ALGORITHM STRUCTURE PREDICTION

To predict "synthetically probable" candidate structures, we apply a genetic algorithm that uses a combination of elitist selection and proportionate selection, with selection probabilities calculated using a Boltzmann fitness function. The genetic algorithm is illustrated diagrammatically in Fig. S5.

Any configuration of anions within a $TiOF_2$ supercell containing N anion sites can be described by a length l vector, with each element set to 1 or 0 according to whether the corresponding anion site is occupied by O or F. For example,

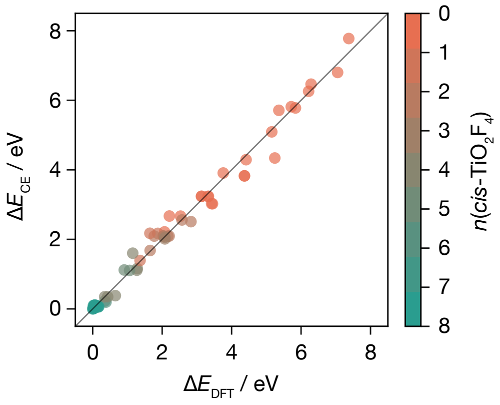

FIG. S3: DFT-calculated relative energies versus corresponding CE-model-predicted relative energies for the 65 $2 \times 2 \times 2$ TiO_2F_4 supercells used for fitting the cluster-expansion model. The diagonal line corresponds to $\Delta E_{DFT} = \Delta E_{CE}$. Points are coloured according to the number of *cis*-TiO_2F_4 coordination octahedra in each structure (up to a maximum of 8).

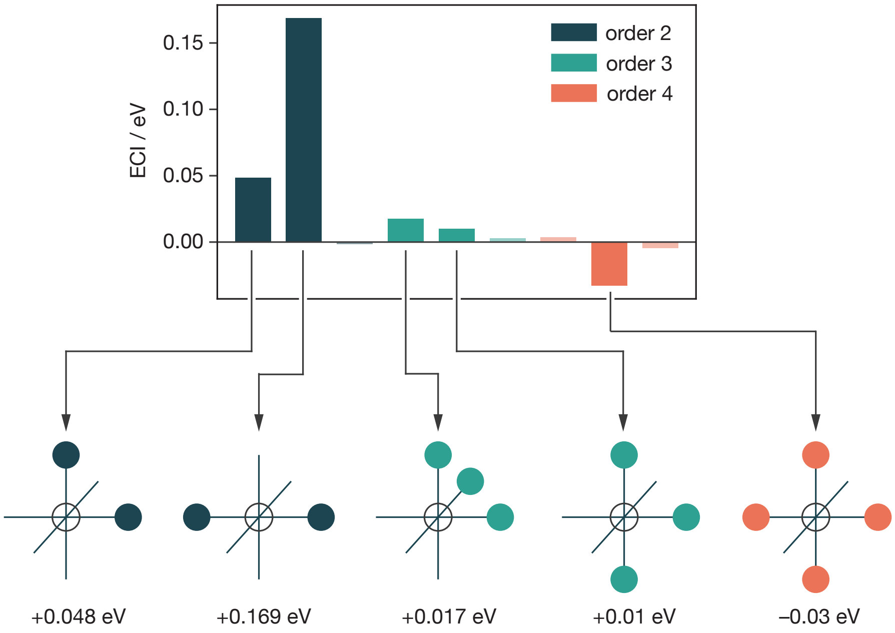

FIG. S4: ECIs for our fitted cluster expansion model for all non-zero ECIs for clusters of order > 1. Five clusters have ECIs > 0.01 eV: for each of these "significant" clusters we show a corresponding representative anion geometry and the ECI value.

for a $4 \times 4 \times 4$ $TiOF_2$ supercell containing 192 anions, any specific anion configuration can be represented by a unique vector of length $l = 192$. Within the genetic algorithm, each configuration vector is considered an individual that exists within a larger population.

We initialised the genetic algorithm by constructing a set of N random vectors $\{\vec{v}_i\}$ with dimension l, where l is the number of anions in the target $TiOF_2$ supercell. To ensure $TiOF_2$ stoichiometry with a F:O ratio of 2:1, each initial vector is constructed such that the L^1 norm of each vector, $\|\vec{v}\|$, is equal to $l/3$. This set of N vectors defines a starting population of N individuals for the genetic algorithm. The genetic algorithm consists of multiple iterations of a series of steps that use these N individuals to generate a new set of N' individuals with different combinations of 1 and 0 elements; i.e., these correspond to new configurations of anions within the $TiOF_2$ supercell. At each iteration of the

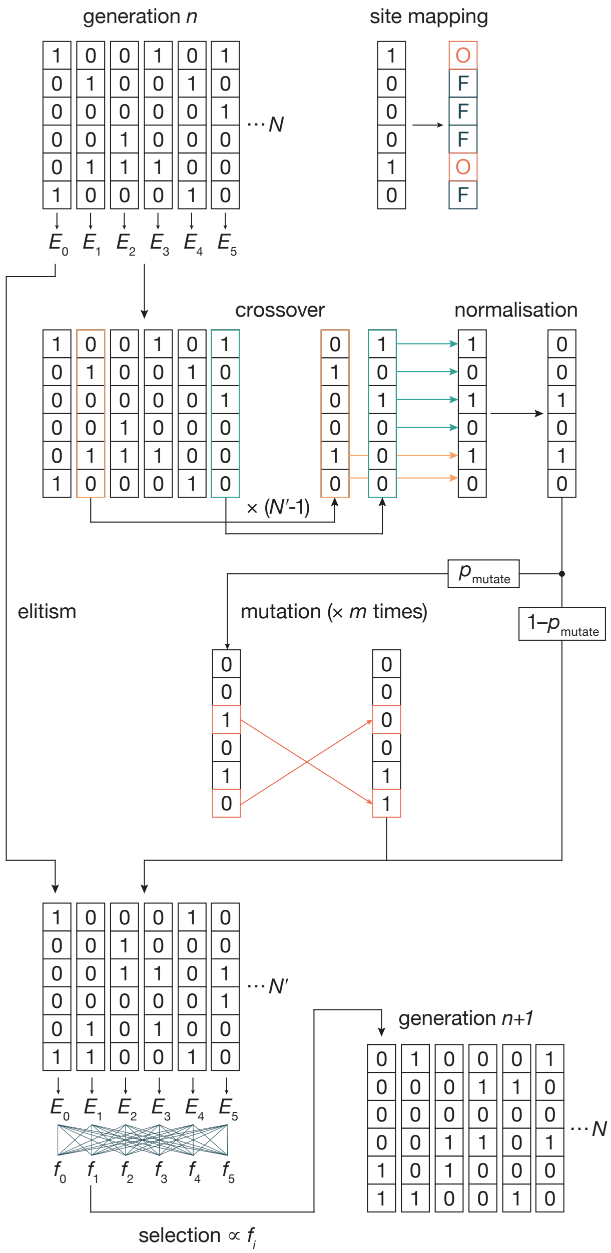

FIG. S5: Diagrammatic representation of the genetic algorithm used to generate probable partially-disordered $TiOF_2$ supercells

genetic algorithm, we select N individuals from this pool of N' candidates based on a Boltzmann-weighted fitness function. These new N individuals form the starting population for the next iteration of the genetic algorithm. By iterating over multiple generations, the genetic algorithm selects for low-energy anion configurations with a Boltzmann probability distribution that is parameterised by our choice of effective temperature.

At each iteration of the genetic algorithm, we first compute the energy, E_i, of each individual, $\vec{v}_i$ in the current population using our $TiOF_2$ cluster expansion Hamiltonian. The individual with the lowest energy is automatically included in the selection pool (elitist selection). We then randomly select $N-1$ pairs of individuals as "parents" that are used to generate new "child" individuals. For each pair of parents, we first apply a crossover operation to construct a new child vector, by selecting each element at random from one of the parents. Because each element in this child vector is selected independently, the resulting vector, $\vec{v}_j$, may not preserve the desired $TiOF_2$ stoichiometry. We therefore apply a normalisation procedure that performs randomised $0 \leftrightarrow 1$ swaps until $\|\vec{v}_j\| = l/3$. We then also

apply a mutation operation with probability $p = 0.15$ that swaps m random pairs of elements of the vector $\vec{v}_j$, where m is a random integer between 1 and 10. The mutation operation preserves the number of 0 and 1 elements so does not require normalisation.

Repeating this procedure N' times gives a pool of N' candidate individuals: one individual corresponds to the lowest energy configuration from the parent generation and the other $N' - 1$ individuals are new configurations that have been generated by "breeding" pairs of individuals from the parent generation. We then apply Boltzmann selection to choose N individuals from this pool to use as the input population for the next iteration of the genetic algorithm. For the Boltzmann selection we calculate the energies of all N' candidates in the pool. We then select without replacement N individuals with a probability given by

$$f_i = \frac{\exp\left(-\Delta E_i/kT\right)}{\sum_i \exp\left(-\Delta E_i/kT\right)}, \tag{S2}$$

where ΔE_i is the energy of each individual with respect to the lowest energy individual in the pool, k is the Boltzmann constant, and T is an effective temperature that sets the selection pressure towards low energy configurations. For the structures reported in the main text, we use an effective selection temperature of $T = 750\,\mathrm{K}$.

S5. STRUCTURAL ANALYSIS OF THE GA-PREDICTED $4 \times 4 \times 4$ SUPERCELL MODELS

Table S1 lists cell parameters and cell volumes for the four $4 \times 4 \times 4$ structures predicted using the genetic algorithm (GA) scheme described above. These structures were fully optimised using DFT (CP2K; PBE + DFT-D3).

TABLE S1: Cell parameters, a, b, c (Å) and α, β, γ (degree), and cell volume, V (Å^3), of the GA-predicted structures 1–4 before and after full optimization; ratio between the fully-optimised structure cell volume and the experimental cell volume.

Structure	initial		full optimisation							
	a	V	a	b	c	α	β	γ	V	V/V_{expt}
1	15.258	3552.1	15.045	15.069	15.056	90.07	90.26	90.04	3413.4	0.966
2	15.426	3670.8	15.343	15.263	15.409	89.95	90.00	90.04	3608.5	1.021
3	15.264	3556.2	15.020	15.022	15.066	90.03	90.03	89.32	3399.1	0.962
4	15.426	3671.0	15.302	15.386	15.307	89.99	90.03	89.96	3603.8	1.020

The four GA-predicted structures exhibit different cell volumes, with structures 1 and 3 having smaller volumes than structures 2 and 4, and show a small variation in their composition of TiX_6 (X = O, F) units (Fig. S6; structures 1–3 have identical compositions, while structure 4 has a slightly lower TiO_2F_4 content.

Additional structural data for the four GA-predicted $4 \times 4 \times 4$ structures are presented in Tables S2 to S4 and Fig. S7. Table S2 summarises the Ti–F and Ti–O bond lengths, as well as the ×–F and ×–O distances, where × represents the centroid of each F_6 octahedron. Table S3 gives data for the nearest-neighbour F–F, F–O, and O–O distances. Table S4 gives data for the average distortions of X–Ti–X and X–×–X angles between adjacent and opposite Ti–X bonds (where X = O or F) and the corresponding X–×–X angles between adjacent and opposite ×–X vectors. This table also presents the average Ti–× off-centre displacements, and average Ti–F–Ti and Ti–O–Ti angles. Fig. S7 shows calculated radial distribution functions for Ti–X, Ti–Ti, and X–X pairs, summed over all four GA-predicted structures.

The Ti–F and Ti–O bond lengths (Table S2) reveal that, on average, Ti–F bonds are longer (1.987 Å) and Ti–O bonds are shorter (1.811 Å) than the mean Ti—X distance. This mean bond-length difference is consistent with the bond-length and iCOHP analyses presented in the main manuscript for the $2 \times 2 \times 2$ supercell dataset (Fig. 4), which predict that anion configurations that allow shorter Ti–O bonds are energetically favoured due to a corresponding increase in net Ti–X bond strength.

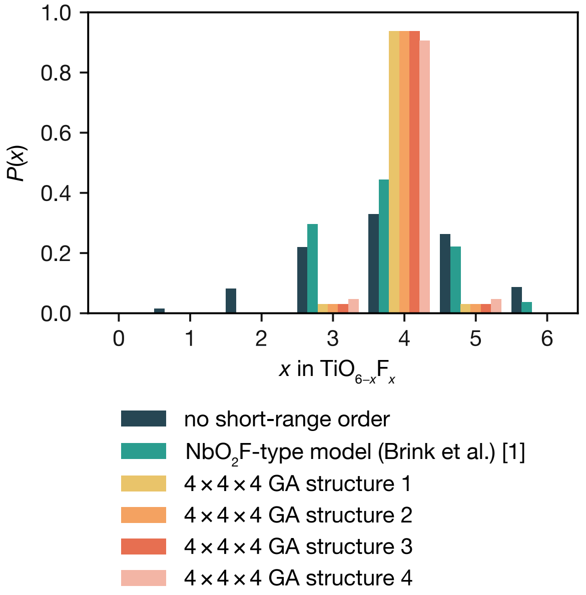

FIG. S6: Distribution of $TiO_{6-x}F_x$ coordination octahedra in ReO_3-type $TiOF_2$ for different structural models: (a) no short-range order (anions are fully uncorrelated); (b) A hypothetical model with intermediate-range [O–F–F–O] ordering along ⟨001⟩ columns, analogous to the structure for NbO_2F proposed by Brink *et al.* [2]; (c–f) Four $4 \times 4 \times 4$ GA-predicted supercells.

TABLE S2: Maximum (d_{max}), minimum (d_{min}), mean ($\langle d \rangle$), and mean absolute deviation from the mean ($\langle |d - \langle d \rangle| \rangle$) for Ti–F, Ti–O, ×–F, and ×–O distances (Å), where × is the centroid of each F_6 anion octahedron, in GA-predicted structures 1–4.

Structure	Ti–F				Ti–O			
	d_{max}	d_{min}	$\langle d \rangle$	$\langle \lvert d - \langle d \rangle \rvert \rangle$	d_{max}	d_{min}	$\langle d \rangle$	$\langle \lvert d - \langle d \rangle \rvert \rangle$
1	2.162	1.879	1.989	0.056	1.909	1.740	1.810	0.033
2	2.171	1.864	1.985	0.057	1.934	1.725	1.812	0.045
3	2.136	1.858	1.988	0.050	1.890	1.750	1.810	0.025
4	2.158	1.878	1.984	0.053	1.917	1.733	1.811	0.034
Average	2.157	1.870	1.987	0.054	1.913	1.737	1.811	0.034
Structure	×–F				×–O			
	d_{max}	d_{min}	$\langle d \rangle$	$\langle \lvert d - \langle d \rangle \rvert \rangle$	d_{max}	d_{min}	$\langle d \rangle$	$\langle \lvert d - \langle d \rangle \rvert \rangle$
1	1.956	1.850	1.912	0.019	1.983	1.896	1.939	0.015
2	1.958	1.841	1.907	0.020	1.995	1.906	1.942	0.016
3	1.970	1.849	1.911	0.020	1.977	1.897	1.939	0.013
4	1.969	1.831	1.907	0.019	1.990	1.906	1.941	0.013
Average	1.963	1.843	1.909	0.020	1.987	1.901	1.940	0.014

Interestingly, the ×–F and ×–O octahedron-centroid to anion distances are much more similar than the Ti–F and Ti–O distances, with the average ×–F distance (1.909 Å) slightly shorter than the average ×–O distance (1.940 Å). The close similarity between ×–F and ×–O distances indicates that the geometry of the anion substructure is closer to an ideal ReO_3-type fcc lattice than might be expected based solely on the large difference in Ti–F and Ti–O distances, and suggests that the principal deviation from ideal octahedral TiX_6 coordination is due to displacement of the Ti cations away from the X_6 octahedra centres.

This interpretation is supported by the nearest-neighbour anion–anion distances (Table S3), with the F–F, F–O, and

TABLE S3: Maximum ($d_{\max}$), minimum ($d_{\min}$), mean ($\langle d \rangle$), and mean absolute deviation from the mean ($\langle |d - \langle d \rangle| \rangle$) for F–F, F–O, and O–O distances (Å) in GA-predicted structures 1–4.

Structure	F–F				F–O				O–O			
	$d_{\max}$	$d_{\min}$	$\langle d \rangle$	$\langle \lvert d - \langle d \rangle \rvert \rangle$	$d_{\max}$	$d_{\min}$	$\langle d \rangle$	$\langle \lvert d - \langle d \rangle \rvert \rangle$	$d_{\max}$	$d_{\min}$	$\langle d \rangle$	$\langle \lvert d - \langle d \rangle \rvert \rangle$
1	2.80	2.60	2.69	0.028	2.82	2.64	2.74	0.023	2.80	2.69	2.74	0.016
2	2.76	2.58	2.68	0.023	2.82	2.66	2.74	0.022	2.79	2.70	2.74	0.015
3	2.78	2.59	2.69	0.025	2.80	2.65	2.74	0.023	2.78	2.71	2.74	0.014
4	2.77	2.56	2.68	0.025	2.83	2.66	2.74	0.023	2.79	2.70	2.74	0.014
Average	2.78	2.58	2.68	0.025	2.82	2.66	2.74	0.023	2.79	2.70	2.74	0.015

O–O distances all showing smaller deviations from their mean values than the Ti–F and Ti–O cation–anion distances, indicating a relatively undistorted anion substructure.

TABLE S4: Mean angular distortions between adjacent ($\langle |\measuredangle XX - 90| \rangle$) and opposite ($\langle 180 - \measuredangle XX \rangle$) Ti–X and ×–X bonds (°), where × is the centroid of a given F_6 octahedron; mean Ti displacement from octahedron centroid $\langle |\times\text{–Ti}| \rangle$ (Å); and mean Ti–F–Ti and Ti–O–Ti angles (°), for GA-predicted structures 1–4.

Structure	X–Ti–X angular distortions		X–×–X angular distortions		Ti displacement	Ti–X–Ti angles	
	$\langle \lvert \measuredangle XX - 90 \rvert \rangle$	$\langle \lvert 180 - \measuredangle XX \rvert \rangle$	$\langle \lvert \measuredangle XX - 90 \rvert \rangle$	$\langle \lvert 180 - \measuredangle XX \rvert \rangle$	$\langle \lvert \times\text{–Ti} \rvert \rangle$	$\langle$Ti–F–Ti$\rangle$	$\langle$Ti–O–Ti$\rangle$
1	4.75	10.17	0.90	1.44	0.20	155.24	157.94
2	5.00	10.33	0.81	1.47	0.21	170.10	172.53
3	4.68	10.15	0.80	1.35	0.20	154.77	157.34
4	4.78	10.25	0.86	1.64	0.20	170.45	172.54
Average	4.80	10.23	0.85	1.47	0.20	162.64	165.09

Analysis of the angular distortions of the TiX_6 octahedra (Table S4), which we characterize using the mean absolute difference between X–Ti–X and X–×–X angles and the corresponding ideal values of 90° for *cis* anion pairs and 180° for *trans* anion pairs in a perfect octahedron, provides further insight into the local structure. The $4 \times 4 \times 4$ structures exhibit large average X–Ti–X angular distortions, but significantly smaller X–×–X angular distortions. This discrepancy further supports the model of a relatively undistorted anion sublattice with significant Ti off-centering. Direct calculation of the ×–Ti distances confirms the large off-centre displacement of Ti, with an average displacement of 0.20 Å.

The large off-center Ti displacement, combined with the average Ti–X bond lengths exceeding $a/2$ (where a is the cubic unit cell parameter), results in compressed and consequently bent Ti–F–Ti and Ti–O–Ti linkages. Generally, the Ti–F–Ti angles are slightly smaller than the Ti–O–Ti angles, likely due to the longer Ti–F bonds. Notably, the most significant difference among the structural models is their average Ti–X–Ti angles, which are smaller for models 1 and 3, and which correlates with the smaller cell volumes for these models (see Table S1).

The calculated radial distribution functions (RDFs) for the Ti–X, Ti–Ti, and X–X pairs, shown in Fig. S7, provide further support for the geometric analysis above, as well as for the peak assignment for the experimental PDF data, as discussed in the main manuscript. First, the average Ti–O nearest-neighbour distance is shorter than the average Ti–F nearest-neighbour distance, as expected from the numerical data in Table S2. We also observe a clear splitting in the Ti–Ti nearest neighbour peak. Both features are consistent with our interpretation of the experimental PDF data in the main manuscript. Second, the nearest-neighbour O–O, O–F, and F–F peaks appear at similar distances, and show a smaller spread in anion–anion distances (narrower peak widths) than for the Ti–O and Ti–F pairs, again, in accordance with the data in Tables S3 and S9.

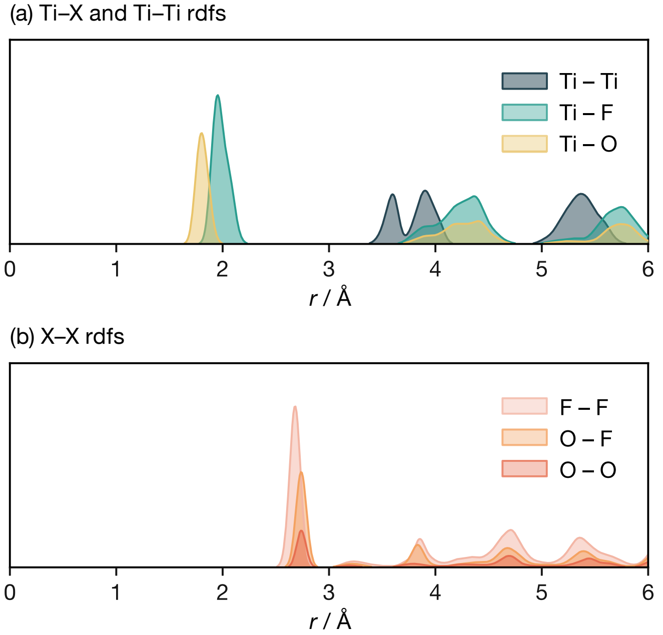

FIG. S7: Ti–X, Ti–Ti, and X–X (X = O, F) radial distribution functions for ReO_3-type $TiOF_2$, calculated from the four $4 \times 4 \times 4$ GA-predicted structural models.

S6. STRUCTURAL ANALYSIS OF THE GA-PREDICTED $6 \times 6 \times 6$ SUPERCELL MODEL

Figs. S8 and S9 present data on the short- and intermediate-range structure in $6 \times 6 \times 6$ $TiOF_2$ supercells for three structural models: a model with fully uncorrelated anions; a model that is analogous to the structural model for NbO_2F proposed by Brink *et al.* [2], with intermediate-range [O–F–F–O] ordering along $\langle 001 \rangle$ columns; and a $6 \times 6 \times 6$ supercell generated using our genetic-algorithm structure-prediction scheme.

Comparing the relative populations of $TiO_{6-x}F_6$ coordination octahedra in each model (Fig. S8), the model with no short-range order follows the expected binomial distribution ($n = 6, p(\mathrm{F}) = \frac{2}{3}$). The NbO_2F-analogue model, after Brink *et al.* [2], gives a narrower distribution of $TiO_{6-x}F_6$ coordination environments, but less than half of the Ti are predicted to have TiO_2F_4 coordination. In contrast, the GA-predicted model contains $\sim 90\,\%$ TiO_2F_4, indicating significantly stronger short-range ordering compared to the Brink NbO_2F model.

The difference between the Brink NbO_2F-type model [2] and our GA-predicted structure is also evident in the distribution of anion orderings along each $\langle 001 \rangle$ column (Fig. S9). The NbO_2F Brink model consists entirely of [O–F–F–O–F–F] column orderings, while the structure obtained using genetic algorithm prediction, based on our DFT-derived cluster expansion model, exhibits a mix of anion orderings along $\langle 001 \rangle$ columns, with [O–F–O–F–F–F] favoured over [O–F–F–O–F–F] (Fig. S9).

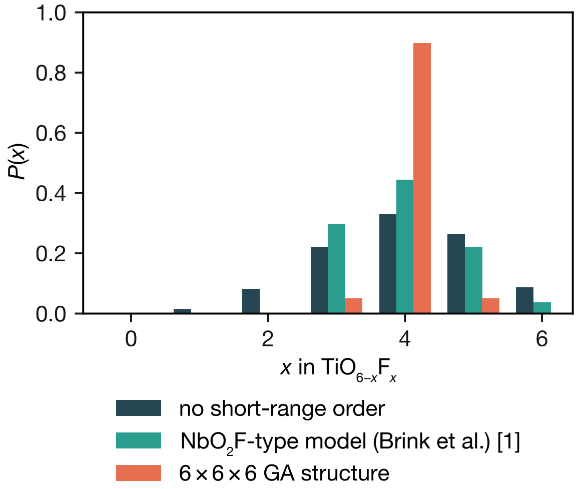

FIG. S8: Distribution of $TiO_{6-x}F_x$ coordination octahedra in ReO_3-type $TiOF_2$ for three structural models: (a) no short-range order (anions are fully uncorrelated); (b) A hypothetical model with intermediate-range [O–F–F–O] ordering along ⟨001⟩ columns, analogous to the structure for NbO_2F proposed by Brink *et al.* [2]; (c) An exemplar $6 \times 6 \times 6$ GA-predicted supercell.

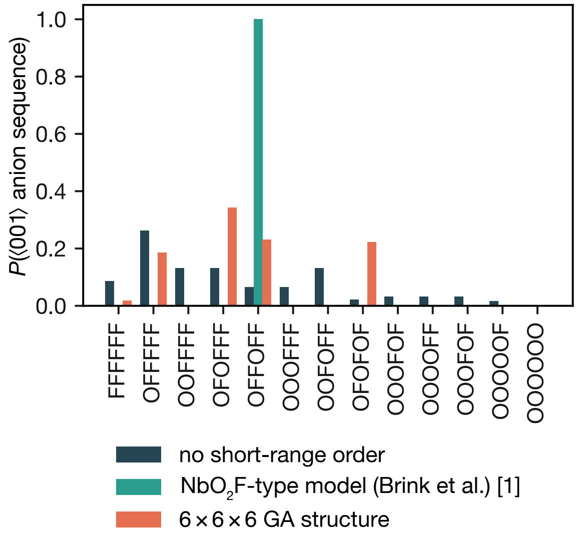

FIG. S9: Distribution of anion orderings along ⟨001⟩ columns in ReO_3-type $TiOF_2$ for three $6 \times 6 \times 6$ supercell models: (a) no short-range order (anions are fully uncorrelated); (b) A hypothetical model with full [O–F–F–O] ordering along ⟨001⟩ columns, analogous to the structure for NbO_2F proposed by Brink *et al.* [2]; (c) An exemplar $6 \times 6 \times 6$ GA-predicted supercell.

S7. CORRELATION BETWEEN CALCULATED $\sigma_{\rm iso}$ AND EXPERIMENTAL $\delta_{\rm iso}$ VALUES FOR ^{19}F IN TITANIUM (OXY)FLUORIDES

A. Haeberlen convention for the shielding and chemical shift NMR parameters

According to the Haeberlen convention [3], the chemical shift tensor can be described by three parameters: the isotropic chemical shift, $\delta_{\rm iso}$, the chemical shift anisotropy, $\delta_{\rm csa}$, and the asymmetry parameter, $\eta_{\rm csa}$. These parameters are related to elements of the chemical shift tensor via $\delta_{\rm iso} = \frac{1}{3}(\delta_{xx} + \delta_{yy} + \delta_{zz})$, $\delta_{\rm csa} = \delta_{zz} - \delta_{\rm iso}$, and $\eta_{\rm csa} = (\delta_{yy} + \delta_{xx})/\delta_{\rm csa}$, with the principal components defined in the sequence $|\delta_{zz} - \delta_{\rm iso}| \geq |\delta_{xx} - \delta_{\rm iso}| \geq |\delta_{yy} - \delta_{\rm iso}|$.

The magnetic shielding tensor can similarly be described by three parameters: the isotropic shielding, $\sigma_{\rm iso}$, the shielding anisotropy, $\sigma_{\rm csa}$, and the asymmetry parameter, $\eta_{\rm csa}$, with these defined as $\sigma_{\rm iso} = \frac{1}{3}(\sigma_{xx} + \sigma_{yy} + \sigma_{zz})$, $\sigma_{\rm csa} = \sigma_{zz} - \sigma_{\rm iso}$, and $\eta_{\rm csa} = (\sigma_{yy} + \sigma_{xx})/\sigma_{\rm csa}$, with the principal components defined in the sequence $|\sigma_{zz} - \sigma_{\rm iso}| \geq |\sigma_{xx} - \sigma_{\rm iso}| \geq |\sigma_{yy} - \sigma_{\rm iso}|$.

B. Details about calculations using the NMR-CASTEP code

DFT periodic calculations of the ^{19}F chemical shielding tensors in TiF_4 [4], using the GIPAW method [5, 6], were performed with the NMR-CASTEP code [7, 8], implemented in the MATERIALS STUDIO 5.0 environment, for the experimental and geometry optimized structures. The Perdew-Burke-Ernzerhof (PBE) functional [9] was employed in the generalized gradient approximation (GGA) for the exchange correlation energy. "On-the-fly" ultrasoft pseudopotentials (USPP) [6] provided in CASTEP were used with core radii of $1.4\,a_0$ for F and $1.8\,a_0$ for Ti, with 2s and 2p valence orbitals for F and 3s, 3p, 4s and 3d valence orbitals for Ti. The wave functions were expanded on a plane-wave basis set with a kinetic energy cutoff of 700 eV. The total energy was converged up to change below 1×10^{-8} eV. The Brillouin zone was sampled using a Monkhorst-Pack grid spacing lower than $0.025\,\text{Å}^{-1}$, corresponding to a k-point mesh of $2 \times 12 \times 5$. Geometry optimized structures were obtained by minimizing the residual forces ($|F|_{\rm max} < 10\,\text{meV}\,\text{Å}^{-1}$) for all atoms, using the Broyden-Fletcher-Goldfarb-Shanno (BFGS) method [10] and keeping symmetry constraints and fixing cell parameters to the experimentally measured values.

C. Previously reported relationships between calculated $\sigma_{\rm iso}$ and experimental $\delta_{\rm iso}$ values for ^{19}F in inorganic fluorides

For ^{19}F in inorganic fluorides, several empirical linear relationships between DFT-calculated $\sigma_{\rm iso}$ values and experimental $\delta_{\rm iso}$ values have previously been proposed [11–32] (see Table S5). These empirical models all follow the general form $\delta_{\rm iso} = a\sigma_{\rm iso} + \sigma_{\rm ref}$. Two different approaches to fitting this linear relationship model are found in the literature: in the simplest approach, the parameter a is fixed to the theoretical value of -1 and only $\sigma_{\rm ref}$ is fitted, giving a model of the form $\delta_{\rm iso} = -\sigma_{\rm iso} + \sigma_{\rm ref}$; alternatively, a and $\sigma_{\rm ref}$ are both fitted, to give a model of the form $\delta_{\rm iso} = a\sigma_{\rm iso} + \sigma_{\rm ref}$. In the case of ^{19}F, the approach of fixing $a = -1$ has been used only once [13], probably because, when using generalized gradient approximation (GGA) exchange-correlation functionals, the absolute values of a determined by full linear regression are often significantly less than 1, and optimising both a and $\sigma_{\rm ref}$, therefore, gives a much better description of the correlation between calculated $\sigma_{\rm iso}$ and experimental $\delta_{\rm iso}$ values [31]. NbF_5 [14] and TiF_4 [25] provide exceptions to this general behaviour: for these materials the values of a obtained from full linear regression are close to the theoretical value of -1. There have also been attempts to correct this shortcoming of GGA functionals to give empirical $\sigma_{\rm iso} \mapsto \delta_{\rm iso}$ fits that are closer to the theoretical $\delta_{\rm iso} = -\sigma_{\rm iso} + \sigma_{\rm ref}$ behaviour [24, 31].

For compounds containing Ti^{IV} atoms, five empirical linear relationships between calculated $\sigma_{\rm iso}$ and experimental $\delta_{\rm iso}$ values have been reported in the literature (Table S5) [22, 26, 27, 29]. Four of these are unsuitable for simulating the ^{19}F NMR spectra of titanium oxyfluorides, for the following reasons:

i. For $K_{16}[Mo_3O_4F_9]_2[TiF_6]_3 \cdot 2\,H_2O$, the Ti-bound F atoms have not been considered [26];
ii. In $[H_2taz]_2 \cdot (Ti_5O_5F_{12})$ and $[H_2gua]_2 \cdot (Ti_5O_5F_{12})$, there is evidence of disorder, which gives correlated deviations from average δ_{iso} and σ_{iso} values [22].
iii. In *trans*-$Zn_3TiF_7(H_2O)_2(taz)_3 \cdot 3\,H_2O$, *trans*-$Zn_3TiF_7(taz)_3$, and *cis*-$Zn_3TiF_7(H_2O)_2(taz)_3 \cdot C_2H_5OH$, the F environments differ (F–Zn_3, F–TiZn, and F–Ti), but the latter two are not experimentally differentiated [27].
iv. In $CaTiF_6(H_2O)_2$, five F atoms are corner-shared with Ca polyhedra, while one F atoms is terminal and H-bonded with water molecules [29].

The fifth previously published $\sigma_{iso} \mapsto \delta_{iso}$ relationship for ^{19}F in compounds containing Ti^{IV} was derived by Murakami *et al.* for TiF_4 [25]. TiF_4 which contains several crystallographically distinct fluoride positions with these F all directly bonded to Ti [4], making it a good reference system for deriving a general empirical relationship between σ_{iso} and δ_{iso} for titanium (oxy)fluorides. The linear relationship between σ_{iso} and δ_{iso} determined by Murakami *et al.* (S10), however, suffers from a number of deficiencies, as discussed in the main manuscript, prompting us to revisit the derivation of a general relationship between σ_{iso} and δ_{iso} values for ^{19}F in titanium (oxy)fluorides.

S8. DERIVATION OF AN EMPIRICAL LINEAR RELATION BETWEEN CALCULATED σ_{iso} AND EXPERIMENTAL δ_{iso} VALUES FOR ^{19}F IN TITANIUM (OXY)-FLUORIDES

Fig. S10(b) shows a plot of calculated σ_{iso} data versus experimental δ_{iso} data for TiF_4, using the σ_{iso} and δ_{iso} data reported by Murakami *et al.* The data are clustered in two groups, corresponding to terminal and bridging F, and these two subsets of fluorine data are shown in more detail in panels S10(a) and S10(c) for terminal and bridging F, respectively. While the data appear to follow an approximately linear relationship, closer examination of the terminal and bridging data shows that the best-fit linear model derived using the complete dataset gives systematic errors for both the terminal and bridging subsets. An improved description for the terminal and bridging fluorine atoms can be obtained by fitting separate linear models each data subset, as shown by the solid lines in panels S10(a) and S10(c). For the previously published data of Murakami *et al.*, fitting two separate linear models gives much better agreement for the bridging F, while the data for the terminal F still show considerable deviation from the corresponding best-fit model.

Fig. S11(a–c) and Fig. S12(a–c) show σ_{iso} versus δ_{iso} ^{19}F data with σ_{iso} values that we have computed using CASTEP [7, 8] (Fig. S11) and VASP [33, 34] (Fig. S12). In both cases, we again find that a single linear model fitted to data for both the terminal and bridging fluorine gives an unsatisfactory description of the bridging F data, and an improved description is obtained by fitting independent linear models to the terminal and bridging F separately. Interestingly, the terminal F data from these DFT calculations are much better described by a linear relationship between σ_{iso} and δ_{iso} than the data reported by Murakami *et al.*, which we attribute to our calculations being performed with better converged settings. In addition to the two datasets presented here (Fig. S11 and Fig. S12), where the σ_{iso} values were computed for TiF_4 geometries with optimisation of atomic positions only (APO), using the PBE exchange-correlation functional, we also performed an atomic-positions-only geometry optimisation of TiF_4 using PBE supplemented by the DFT-D3 dispersion correction of Grimme *et al.* [35], which we expect to give a more accurate description of the columnar structure of TiF_4. Plots of σ_{iso} versus δ_{iso} and σ_{csa} versus δ_{csa} for this VASP-PBE-DFT-D3 dataset (Table S7) is shown in the main manuscript (Fig. 10). For all three sets of newly calculated ^{19}F data, we obtain very good linear correlation between the calculated ^{19}F σ_{iso} values and the experimental ^{19}F δ_{iso} values (Figs. S11 and S12 and Fig. 10). The coefficients of determination, R^2, are close to 1, and the calculated δ_{iso} values predicted these linear relations are close to the corresponding experimental values (Tables S6 and S7): this good linear consistency between our calculated σ_{iso} ^{19}F data and the experimental δ_{iso} ^{19}F NMR data supports our assignment of individual resonances to specific F sites, and provides confidence in the resulting best-fit σ_{iso}-δ_{iso} relationships .

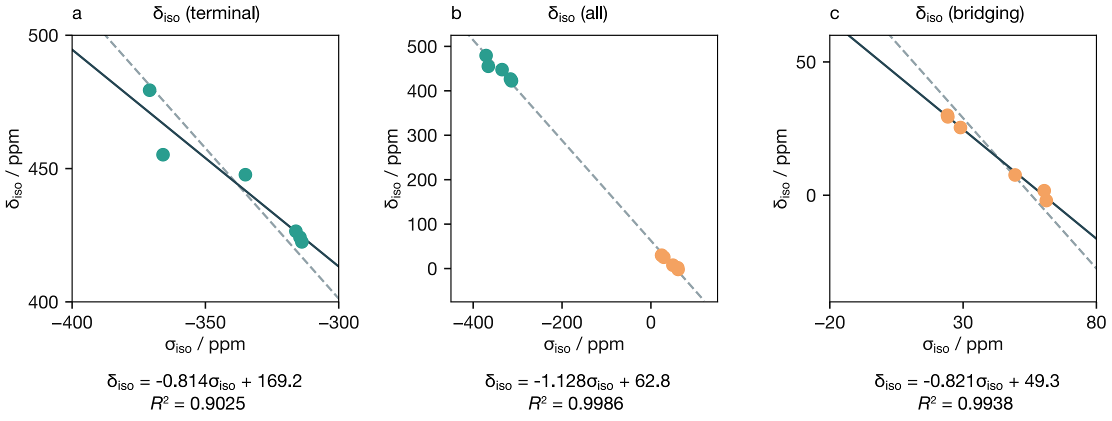

FIG. S10: [19]F experimental δ_{iso} values [25] as a function of calculated σ_{iso} values of TiF_4 as reported by Murakami *et al.* [25]. Panel (b) shows all the data, and corresponding linear-least-squares fit (dashed line). Panels (a) and (c) show the same data, selecting values for terminal F and bridging F only, respectively; each panel shows the original linear model obtained from fitting to the full dataset (dashed lines) and a revised linear model obtained by fitting to the corresponding data subset only (solid lines). Equations for the revised and original linear models are given below the corresponding panels.

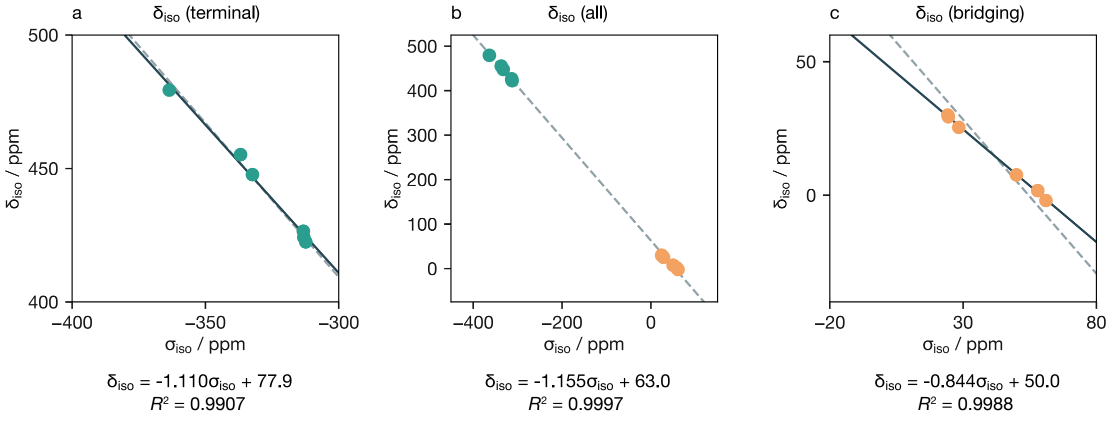

FIG. S11: [19]F experimental δ_{iso} values [25] as a function of calculated σ_{iso} values of TiF_4 using CASTEP. Panel (b) shows all the data, and corresponding linear-least-squares fit (dashed line). Panels (a) and (c) show the same data, selecting values for terminal F and bridging F only, respectively; each panel shows the original linear model obtained from fitting to the full dataset (dashed lines) and a revised linear model obtained by fitting to the corresponding data subset only (solid lines). Equations for the revised and original linear models are given below the corresponding panels. Numerical data for this figure are listed in Table S6.

S9. EFFECT OF DFT CALCULATION METHOD ON ATOMIC POSITIONS FOR TiF_4

In this section we present comparisons of the atomic positions of TiF_4 from experiment [4] and calculated using DFT (optimisation of atomic positions only), performed with CASTEP (APO CASTEP), VASP without the DFT-D3 dispersion corrections (APO VASP), and VASP with the DFT-D3 dispersion correction (APO VASP DFT-D3). Crystallographic Information Files (CIF) of these DFT-optimised structures are available as part of the Supplementary Dataset.

Optimizations with CASTEP, VASP, and VASP with the DFT-D3 dispersion correction led to atomic displacements (Table S8) ranging from 0.026 Å to 0.033 Å, 0.013 Å to 0.051 Å, and 0.010 Å to 0.058 Å for the Ti atoms, respectively.

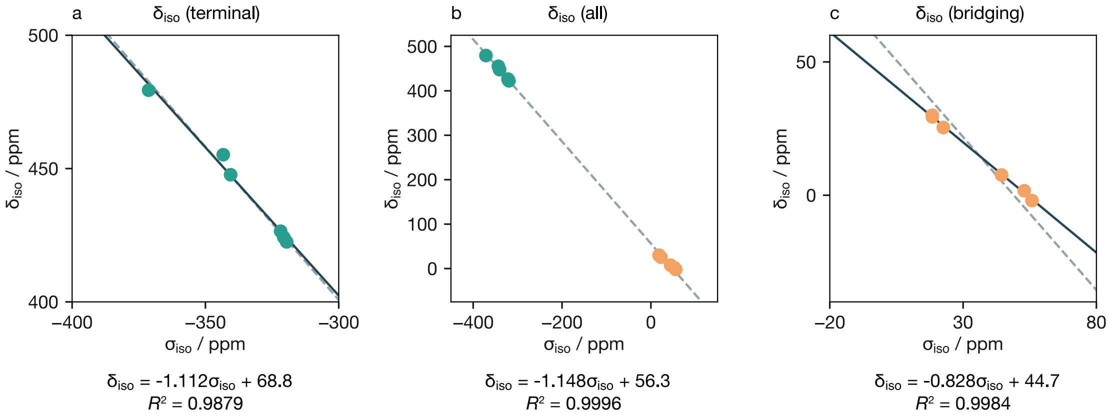

FIG. S12: [19]F experimental δ_{iso} values [25] as a function of calculated σ_{iso} values of TiF_4 using VASP without DFT-D3. Panel (b) shows all the data, and corresponding linear-least-squares fit (dashed line). Panels (a) and (c) show the same data, selecting values for terminal F and bridging F only, respectively; each panel shows the original linear model obtained from fitting to the full dataset (dashed lines) and a revised linear model obtained by fitting to the corresponding data subset only (solid lines). Equations for the revised and original linear models are given below the corresponding panels. Numerical data for this figure are listed in Table S6.

For the F atoms, the displacements ranged from 0.011 Å to 0.071 Å, 0.005 Å to 0.085 Å, and 0.014 Å to 0.096 Å respectively. The positions of the terminal F atoms were most affected by the choice of optimisation method. During optimisation, the Ti–F bond lengths (Tables S9 and Table S10) increased relative to the experimental structure, with a larger increase observed for the terminal (0.038 Å or 0.037 Å on average) compared to the bridging F atoms (0.013 Å or 0.014 Å on average). The radial distortions of the TiF_6 octahedra, which are primarily due to the presence of two types of F atoms at the vertices, slightly decrease (Table S9). However, after optimization, the bond lengths between Ti and the bridging F became more scattered.

Lastly, the angular distortions (in °) of the TiF_6 octahedra, defined as the average of the absolute values of the deviations from ideality, i.e., $|\measuredangle\text{F–Ti–F} - 90°|$ and $180° - \measuredangle\text{F–Ti–F}$, slightly increased (Table S11). Overall, considering that the PBE functional typically overestimates bond lengths, the effects of geometry optimization are moderate and the resulting predicted structure of TiF_4 is quite accurate. It is worth noting that despite yielding quite different atomic displacements, CASTEP and VASP, the latter both with and without DFT-D3, predict very similar Ti–F bond lengths and F–Ti–F angles, with maximum differences of 0.005 Å and 0.27° for adjacent Ti–F bonds, respectively.

S10. ADDITIONAL ^{19}F NMR DATA FOR THE $4 \times 4 \times 4$ GA-PREDICTED STRUCTURAL MODELS

Fig. S13 shows simulated ^{19}F MAS (64 kHz) NMR spectra for ReO_3-type $TiOF_2$ for the four GA-predicted $4 \times 4 \times 4$ supercell models. These spectra were generated using the $\sigma_{\mathrm{iso}} \to \delta_{\mathrm{iso}}$ and $\sigma_{\mathrm{csa}} \to \delta_{\mathrm{csa}}$ relationships derived from fitting DFT calculated σ_{iso} and σ_{csa} values to experimental δ_{iso} and δ_{csa} values for bridging F atoms in TiF_4 [25]. Summary statistics for each simulated spectrum are presented in Table S12.

In the absence of a formal R factor to quantify the agreement between simulated and experimental spectra, we employ two main criteria to designate the “best” structural model: the deviation between the simulated and experimental mean δ_{iso} values, and the spread of δ_{iso} values. The spread of δ_{iso} values is particularly relevant because the ^{19}F chemical shift of a fluoride ion depends primarily on the identity and distance of its nearest-neighbour metal cations. This dependence is approximately exponential with distance, making the spread of δ_{iso} values a good characterization of the distribution of Ti–F bond lengths.

The GA-predicted structure 4, which provides the best fit to the PDF data, also gives a good compromise with respect to the ^{19}F NMR data for both the mean of the predicted δ_{iso} values and their spread. Structure 2 shows a larger predicted deviation between simulated and experimental mean δ_{iso} values ($\delta_{\text{iso}}^{\text{cal}} = 25.4\,\text{ppm}$ versus $\delta_{\text{iso}}^{\text{expt}} = 17.6\,\text{ppm}$). Structures 1 and 3, while having mean δ_{iso} values closer to the experimental value, exhibit larger spreads in δ_{iso} values, indicating a wider distribution of local F environments than observed experimentally.

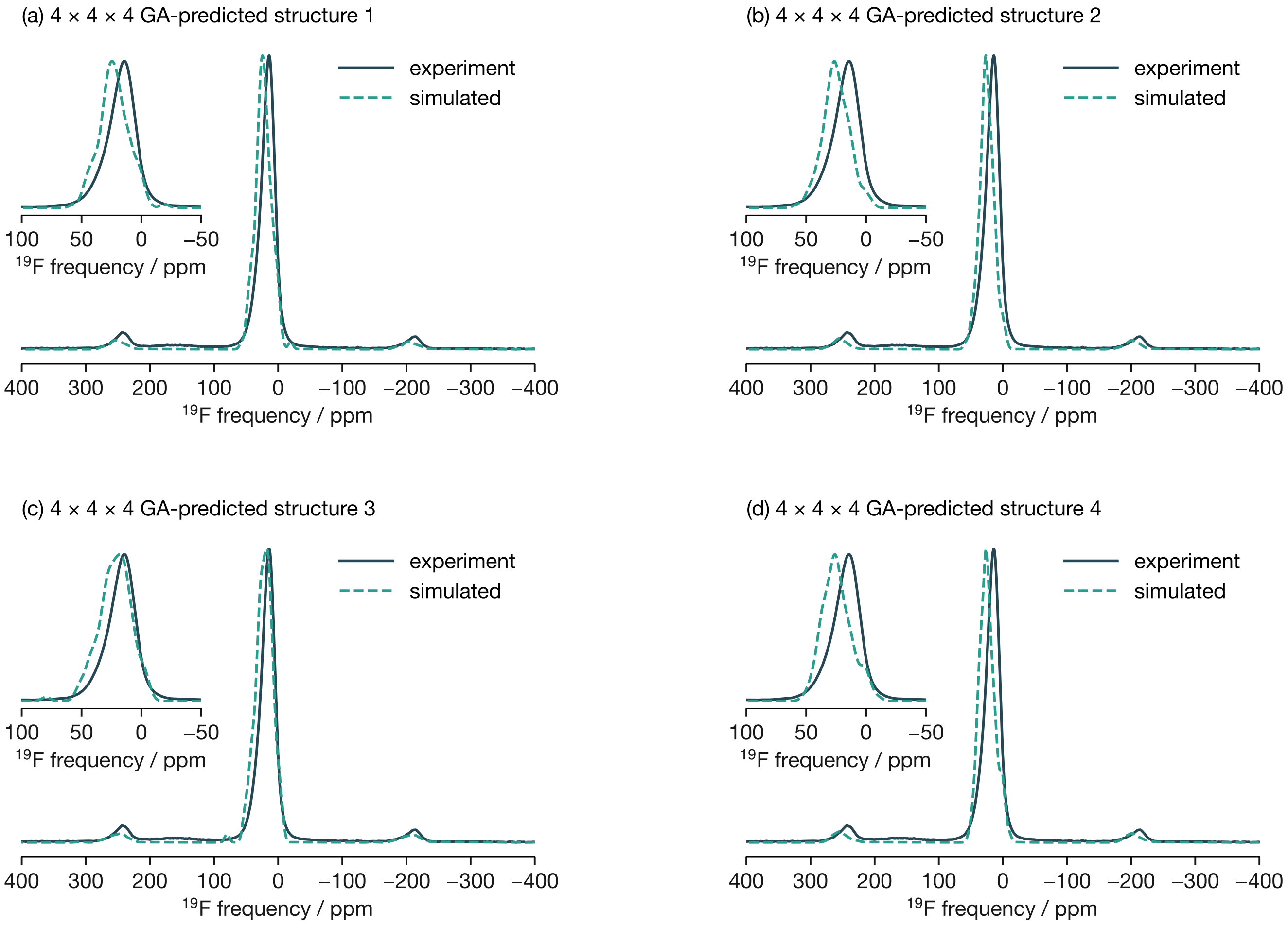

FIG. S13: Comparisons between experimental (solid lines) and simulated (dashed lines) ^{19}F MAS (64 kHz) NMR spectra for ReO_3-type $TiOF_2$ for all four GA-predicted $4 \times 4 \times 4$ supercell models.

SUPPORTING DATA

Data and plotting scripts for all figures, except Fig. S5, are available on GitHub [36]. This repository also includes Cif files for TiF_4 optimised using DFT (atomic positions only), using CASTEP, VASP without DFT-D3, and VASP with DFT-D3.

TABLE S5: Previously reported relationships between calculated σ_{iso} values and experimental δ_{iso} values for ^{19}F in inorganic fluorides and, when reported, relationships between the calculated σ_{csa} values and the experimental δ_{csa} values.

Inorganic fluoride(s)	Equations	Ref.
LiF, NaF, KF, RbF, $KF \cdot 2H_2O$, $RbF \cdot H_2O$, CaF_2, SrF_2, CdF_2, HgF_2, Hg_2F_2, α-PbF_2, α-AlF_3, LaF_3, Na_3AlF_6, $Na_5Al_3F_{14}$, $KAlF_4$, Na_2SiF_6, and two organic fluorides.	$\delta_{iso} = -0.68(\sigma_{iso} - 81.39)$<br>$\delta_{csa} = -0.68\sigma_{csa}$	11
MF (M = Li, Na, K, Rb), MF_2 (M = Ca, Sr, Ba), MF_3 (M = Sc, Y, La), CdF_2, HgF_2, Hg_2F_2, α-PbF_2, α-AlF_3, $Na_5Al_3F_{14}$, ZnF_2, GaF_3, InF_3, $BaLiF_3$, β-−$BaAlF_5$, and $Ba_3Al_2F_{12}$	$\delta_{iso} = -0.80(3)\sigma_{iso} + 89(9)$	12
NaF, CaF_2, Na_2SiF_6, $Ca_4(Si_2O_7)F_2$,<br>Fluoroapatite $Ca_5(PO_4)_3F$	$\delta_{iso} = -\sigma_{iso} + 170.5$<br>$\delta_{iso} = -0.963\sigma_{iso} + 159.6$	13
NbF_5<br>TaF_5	$\delta_{iso} = -1.028(6)\sigma_{iso} + 100.4(8)$<br>$\delta_{iso} = -0.777(11)\sigma_{iso} + 86.2(1.2)$	14
MF (M = Li, Na, K, Rb, Cs), MF_2 (M = Ca, Sr, Ba)<br>MF (M = Li, Na, K, Rb, Cs, Tl), MF_2 (M =Mg, Ca, Sr, Ba), MF_3 (M = Al, Ga, In)	$\sigma_{iso} = -1.26(0.038)\delta_{iso} + 123.2$<br>$\sigma_{iso} = -0.964(0.036)\delta_{iso} + 191.2$<br>$\sigma_{iso} = -0.817(0.036)\delta_{iso} + 236.3$<br>$\sigma_{iso} = -1.05(0.044)\delta_{iso} + 184.5$	31
MF (M = Li, Na, K, Rb), MF_2 (M = Ca, Sr, Ba), MF_3 (M = Sc, Y, La), α-AlF_3, β-AlF_3, η-AlF_3, GaF_3, TlF−II, TlF−I	$\delta_{iso} = -0.795(15)\sigma_{iso} + 89(4)$	15
Fluorinated imidazolium salts<br>$[C_6H_4(CH_2(C_4H_6N_2)_2]^{2+}\,2\,[A]^-$ (A = BF_4, PF_6)	$\delta_{iso} = -0.704(\sigma_{iso} - 105.1)$	16
BaF_2, β-$BaAlF_5$, and $Ba_5Al_3F_{19}$	$\delta_{iso} = -0.87\sigma_{iso} + 117$	17
MF (M = Li, Na, K, Rb, Cs, Tl), MF_2 (M = Mg, Ca, Sr, Ba), MF_3 (M = Al, Ga, In)	$\delta_{iso} = -0.7964(0.0250)\sigma_{iso} + 86.47(7.08)$<br>$\delta_{iso} = -0.8056(0.02570)\sigma_{iso} + 87.76(7.22)$<br>$\delta_{iso} = -0.8429(0.0337)\sigma_{iso} + 101.64(9.54)$	32
ScF_3, Na_3ScF_6, Li_3ScF_6, $NaScF_4$, KSc_2F_7, $K_5Sc_3F_{14}$	$\delta_{iso} = -0.589\sigma_{iso} + 20.24$	18
M_2ZrF_6 (M = Li, K, Rb, Cs), K_3ZrF_7, Li_4ZrF_8, $Na_5Zr_2F_{13}$	$\delta_{iso} = -0.795(38)\sigma_{iso} + 82(3)$	19
ThF_4	$\delta_{iso} = -0.938\sigma_{iso} + 77.1$<br>$\delta_{csa} = -0.722\sigma_{csa}$	20
$UO_2F_2 \cdot 1.57\,H_2O$	$\delta_{iso} = -0.4988(\sigma_{iso} + 11.34)$	21
$[H_2taz]_2 \cdot (Ti_5O_5F_{12})$, $[H_2gua]_2 \cdot (Ti_5O_5F_{12})$	$\delta_{iso} = -0.97(9)\sigma_{iso} + 67(5)$	22
KF, γ-K_3YF_6, K_2YF_5, α-KY_3F_{10}, and YF_3	$\delta_{iso} = -0.743(22)\delta_{iso} + 68.5(4.5)$	23
LiF, NaF, KF, CsF, BaF_2	$\sigma_{iso} = -1.233\delta_{iso} + 127.7$<br>$\sigma_{iso} = -1.177\delta_{iso} + 126.0$<br>$\sigma_{iso} = -1.014\delta_{iso} + 192.3$<br>$\sigma_{iso} = -1.005\delta_{iso} + 183.1$	24
TiF_4	$\delta_{iso} = -1.13(1)\sigma_{iso} + 63(3)$<br>$\delta_{csa} = -0.94(20)\sigma_{csa}$	25
$K_5[Mo_3O_4F_9] \cdot 3\,H_2O$, $K_5[Mo_3O_4F_9] \cdot 2\,H_2O$, $K_{16}[Mo_3O_4F_9]_2[TiF_6]_3 \cdot 2\,H_2O$	$\delta_{iso} = -0.824(26)\sigma_{iso} + 66(6)$	26

TABLE S5: Previously reported relationships between calculated $\sigma_{\rm iso}$ values and experimental $\delta_{\rm iso}$ values for ^{19}F in inorganic fluorides (continued).

trans-$Zn_3TiF_7(H_2O)_2(taz)_3 \cdot 3\,H_2O$,<br>*trans*-$Zn_3TiF_7(taz)_3$,<br>*cis*-$Zn_3TiF_7(H_2O)_2(taz)_3 \cdot C_2H_5OH$	$\delta_{\rm iso} = -0.82(1)\sigma_{\rm iso} + 92(3)$	27
Rb_3ScF_6	$\delta_{\rm iso} = -0.730\sigma_{\rm iso} + 70.17$	28
$CeTiF_6(H_2O)_2$	$\delta_{\rm iso} = -0.612(20)\sigma_{\rm iso} + 100.6(1.1)$<br>$\delta_{\rm csa} = -0.945\sigma_{\rm csa}$	29

TABLE S6: Experimental ^{19}F $\delta_{\rm iso}$ (ppm) [25], calculated ^{19}F $\sigma_{\rm iso}$ (ppm) with CASTEP and VASP from the APO structures of TiF_4, calculated ^{19}F $\delta_{\rm iso}$ (ppm) values ($\delta_{\rm iso,cal}$), absolute values of the differences between experimental and calculated $\delta_{\rm iso}$ values (Δ). The $\delta_{\rm iso,cal}$ values were calculated using the distinct best-fit linear relationships for terminal (Fig. S11(a) and Fig. S12(a)) and bridging (Fig. S11(c) and Fig. S12(c)) F atoms, respectively. Data for bridging F atoms are shown in italics. In bold, mean Δ values for bridging and terminal F atoms.

		CASTEP				VASP			
$\delta_{\rm iso}$	F site	$\sigma_{\rm iso}$	$\delta_{\rm iso,cal}$	Δ	$\langle\Delta\rangle$	$\sigma_{\rm iso}$	$\delta_{\rm iso,cal}$	Δ	$\langle\Delta\rangle$
−2.0	*F5*	*61.1*	*−1.5*	*0.5*		*55.8*	*−1.5*	*0.5*	
1.7	*F12*	*58.0*	*1.0*	*0.7*		*52.9*	*0.9*	*0.8*	
7.6	*F9*	*50.0*	*7.8*	*0.2*		*44.4*	*7.9*	*0.3*	
25.4	*F2*	*28.4*	*26.0*	*0.6*		*22.6*	*26.0*	*0.6*	
29.4	*F6*	*24.5*	*29.3*	*0.1*		*18.5*	*29.4*	*0.0*	
30.0	*F1*	*24.3*	*29.5*	*0.5*	***0.4***	*18.4*	*29.4*	*0.6*	***0.5***
422.5	F7	−312.4	424.6	2.1		−319.5	424.1	1.6	
424.2	F3	−313.1	424.5	1.2		−320.6	425.3	1.1	
426.5	F11	−313.3	425.6	0.9		−321.8	426.6	0.1	
447.7	F10	−332.4	446.8	0.9		−340.6	447.5	0.2	
455.2	F8	−336.8	451.8	3.4		−343.3	450.6	4.6	
479.4	F4	−363.6	481.5	2.1	**1.8**	−371.3	481.7	2.3	**1.7**

TABLE S7: Experimental TiF_4 ^{19}F $\delta_{\rm iso}$ and $\delta_{\rm csa}$values [25]; ^{19}F $\sigma_{\rm iso}$ and $\sigma_{\rm csa}$ values, calculated with VASP from our APO DFT-D3 optimised structure; calculated ^{19}F $\delta_{\rm iso}$ ($\delta_{\rm iso,cal}$) and $\delta_{\rm csa}$ ($\delta_{\rm csa,cal}$) values (ppm), obtained using our best fit linear model for $\delta_{\rm iso} = a\sigma_{\rm iso} + \sigma_{\rm ref}$ (Fig. 10); absolute values of the differences between experimental and calculated $\delta_{\rm iso}$values (Δ), $\delta_{\rm iso,cal}$ and $\delta_{\rm csa,cal}$ values of the terminal and bridging F atoms deduced from the relationships between $\sigma_{\rm iso}$ and $\delta_{\rm iso}$ values (Fig. 10(b,c)), on the one hand, and between $\sigma_{\rm csa}$ and $\delta_{\rm csa}$ values (Fig. 10(f,e)), on the other hand, for terminal and bridging F atoms, respectively. Upper and lower groups of fluorine correspond to bridging and terminal fluorine atoms, respectively. Data for bridging F atoms are shown in italics. Average Δ values for bridging and terminal F atoms are shown in bold.

$\delta_{\rm iso}$	F site	$\sigma_{\rm iso}$	$\delta_{\rm iso,cal}$	Δ	$\langle\Delta\rangle$	$\delta_{\rm csa}$	$\sigma_{\rm csa}$	$\delta_{\rm csa,cal}$
−2.0	*F5*	*55.2*	*−1.7*	*0.3*		*−146.7*	*204.0*	*−136.8*
1.7	*F12*	*52.0*	*1.0*	*0.7*		*−138.3*	*210.8*	*−141.4*
7.6	*F9*	*43.7*	*7.9*	0.3		*−146.5*	*213.8*	*−143.4*
25.4	*F2*	*21.9*	*25.9*	*0.5*		*−171.3*	*260.6*	*−174.8*
29.4	*F6*	*17.8*	*29.3*	*0.1*		*−179.5*	*264.2*	*−177.2*
30.0	*F1*	*17.6*	*29.5*	*0.5*	***0.4***	*−171.0*	*264.6*	*−177.5*
422.5	F7	−320.1	424.5	2.0		−562.3	606.8	−556.8
424.2	F11	−320.5	424.8	0.6		−554.1	611.8	−561.4
426.5	F3	−322.5	427.1	0.6		−545.9	613.3	−562.8
447.7	F10	−341.3	448.1	0.4		−594.5	639.0	−586.4
455.2	F8	−342.8	449.8	5.4		−586.5	633.2	−581.1
479.4	F4	−371.5	481.8	2.4	**1.9**	−611.1	660.5	−606.5

TABLE S8: Atomic displacements (Å) after optimisation of the atomic positions of TiF_4 from calculations using CASTEP, VASP without DFT-D3, and VASP with DFT-D3. Upper and lower groups of fluorine correspond to bridging and terminal fluorine atoms, respectively.

Atom	CASTEP	VASP	VASP DFT-D3
Ti1	0.033	0.051	0.058
Ti2	0.026	0.013	0.010
Ti3	0.026	0.039	0.046
F1	0.020	0.037	0.044
F2	0.011	0.020	0.024
F5	0.029	0.050	0.053
F6	0.021	0.005	0.014
F9	0.011	0.033	0.038
F12	0.023	0.019	0.023
F3	0.071	0.059	0.058
F4	0.065	0.080	0.087
F7	0.053	0.085	0.096
F8	0.060	0.068	0.067
F10	0.070	0.085	0.088
F11	0.054	0.060	0.067

TABLE S9: Experimental and DFT-optimised Ti–F bond lengths (Å) for TiF_4 for calculations performed with CASTEP and VASP, both with and without DFT-D3.

Ti	F	multiplicity	experiment [25]	CASTEP	VASP	VASP DFT-D3
Ti1	F10	1	1.715	1.755	1.755	1.755
	F7	1	1.725	1.759	1.759	1.759
	F1 (b)	2	1.932	1.933	1.933	1.933
	F9 (b)	1	1.960	1.977	1.976	1.980
	F5 (b)	1	1.979	2.007	2.008	2.011
	mean ± s.d.	–	1.874 ± 0.110	1.894 ± 0.100	1.894 ± 0.100	1.895 ± 0.101
Ti2	F8	1	1.716	1.752	1.752	1.753
	F3	1	1.720	1.758	1.758	1.757
	F6 (b)	2	1.932	1.932	1.933	1.933
	F12 (b)	1	1.965	1.982	1.983	1.984
	F9 (b)	1	1.977	2.004	2.007	2.009
	mean ± s.d.	–	1.873 ± 0.111	1.893 ± 0.101	1.894 ± 0.102	1.895 ± 0.102
Ti3	F4	1	1.704	1.744	1.744	1.745
	F11	1	1.719	1.760	1.760	1.760
	F2 (b)	2	1.933	1.934	1.935	1.935
	F5 (b)	1	1.967	1.992	1.990	1.993
	F12 (b)	1	1.974	2.010	2.011	2.012
	mean ± s.d.	–	1.872 ± 0.114	1.896 ± 0.105	1.896 ± 0.106	1.897 ± 0.106

TABLE S10: Experimental and DFT-optimised mean F–Ti bond lengths for terminal (F_t) and bridging (F_b) fluorine atoms in TiF_4.

	experimental [25]	CASTEP	VASP	VASP DFT-D3
$\langle F_t{-}{-}Ti \rangle$	1.717 ± 0.006	1.755 ± 0.005	1.755 ± 0.005	1.754 ± 0.005
$\langle F_b{-}{-}Ti \rangle$	1.951 ± 0.020	1.964 ± 0.032	1.965 ± 0.033	1.965 ± 0.033

TABLE S11: Experimental and DFT-optimised average distortion of F–Ti–F angles (°) between adjacent and opposite Ti–F bonds in TiF_4.

		experimental [25]	CASTEP	VASP	VASP DFT-D3
Ti1	adjacent	4.21	4.59	4.62	4.66
	opposite	9.92	10.41	10.42	10.58
Ti2	adjacent	4.10	4.36	4.38	4.46
	opposite	9.62	9.95	9.92	10.04
Ti3	adjacent	4.15	4.27	4.34	4.38
	opposite	9.71	9.90	9.97	10.06

TABLE S12: Mean, minimum, maximum, and standard deviation of calculated ^{19}F σ_{iso} and δ_{iso} values for the VASP DFT-D3 fully-optimised GA-predicted $4 \times 4 \times 4$ structural models, calculated using the relationship $\delta_{\mathrm{iso}} = -0.830\sigma_{\mathrm{iso}} + 44.1$, established from fitting DFT calculated σ_{iso} values to experimental δ_{iso} values for bridging F atoms in TiF_4 [25] (DFT σ_{iso} data calculated for the experimental cell geometry and only atomic-positions optimised, with PBE + DFT-D3).

	$\langle\sigma_{\mathrm{iso}}\rangle$	$\langle\delta_{\mathrm{iso,cal}}\rangle$	min σ_{iso}	max $\delta_{\mathrm{iso,cal}}$	max σ_{iso}	min $\delta_{\mathrm{iso,cal}}$	s.d. σ_{iso}	s.d. $\delta_{\mathrm{iso,cal}}$
Model 1	25.4	23.1	−15.1	56.7	76.4	−19.3	15.4	12.8
Model 2	22.6	25.4	−16.3	57.6	58.1	−4.1	13.6	11.2
Model 3	25.9	22.6	−43.3	80.0	62.0	−7.3	16.8	14.0
Model 4	23.7	24.5	−9.8	52.3	65.0	−9.8	14.7	12.2

[1] B. J. Morgan, bsym: A basic symmetry module, J. Open Source Soft. **2**, 370 (2017).

[2] F. J. Brink, R. L. Withers, and L. Norén, An electron diffraction and crystal chemical investigation of oxygen/fluorine ordering in niobium oxyfluoride, NbO_2F, J. Sol. Stat. Chem **166**, 73 (2002).

[3] U. Haeberlen, Advances in magnetic resonance, in *High Resolution NMR in Solids Selective Averaging* (Academic Press, 1976).

[4] H. Bialowons, M. Müller, and B. G. Müller, Titantetrafluorid—eine überraschend einfache kolumnarstruktur, Z. Anorg. Allg. Chem. **621**, 1227 (1995).

[5] C. J. Pickard and F. Mauri, All-electron magnetic response with pseudopotentials: NMR chemical shifts, Phys. Rev. B **63**, 245101 (2001).

[6] J. R. Yates, C. J. Pickard, and F. Mauri, Calculation of NMR chemical shifts for extended systems using ultrasoft pseudopotentials, Phys. Rev. B **76**, 024401 (2007).

[7] M. D. Segall, P. J. D. Lindan, M. J. Probert, C. J. Pickard, P. J. Hasnip, S. J. Clark, and M. C. Payne, First-principles simulation: ideas, illustrations and the CASTEP code, J. Phys.: Condens. Matter **14**, 2717 (2002).

[8] S. J. Clark, M. D. Segall, C. J. Pickard, P. J. Hasnip, M. I. J. Probert, K. Refson, and M. C. Payne, First principles methods using CASTEP, Z. Krist. — Cryst. Mater. **220**, 567 (2005).

[9] J. P. Perdew, K. Burke, and M. Ernzerhof, Generalized gradient approximation made simple, Phys. Rev. Lett. **77**, 3865 (1996).

[10] B. G. Pfrommer, M. Côté, S. G. Louie, and M. L. Cohen, Relaxation of crystals with the quasi-Newton method, J. Comp. Phys. **131**, 233–240 (1997).

[11] J. M. Griffin, J. R. Yates, A. J. Berry, S. Wimperis, and S. E. Ashbrook, High-resolution ^{19}F MAS NMR spectroscopy: Structural disorder and unusual J couplings in a fluorinated hydroxy-silicate, J. Am. Chem. Soc. **132**, 15651 (2010).

[12] A. Sadoc, M. Body, C. Legein, M. Biswal, F. Fayon, X. Rocquefelte, and F. Boucher, NMR parameters in alkali, alkaline earth and rare earth fluorides from first principle calculations, Phys. Chem. Chem. Phys. **13**, 18539 (2011).

[13] A. Pedone, T. Charpentier, and M. C. Menziani, The structure of fluoride-containing bioactive glasses: New insights from first-principles calculations and solid state NMR spectroscopy, J. Mater. Chem. **22**, 12599 (2012).

[14] M. Biswal, M. Body, C. Legein, A. Sadoc, and F. Boucher, NbF_5 and TaF_5: Assignment of ^{19}F NMR resonances and chemical bond analysis from GIPAW calculations, J. Sol. Stat. Chem. **207**, 208–217 (2013).

[15] A. Sadoc, M. Biswal, M. Body, C. Legein, F. Boucher, D. Massiot, and F. Fayon, NMR parameters in column 13 metal fluoride compounds (AlF_3, GaF_3, InF_3 and TlF) from first principle calculations, Sol. Stat. Nucl. Mag. Res. **59–60**, 1–7 (2014).

[16] M. Neouze, M. Kronstein, M. Litschauer, M. Puchberger, C. Coelho, C. Bonhomme, C. Gervais, and F. Tielens, Exploring the molecular structure of imidazolium–silica-based nanoparticle networks by combining solid-state NMR spectroscopy and first-principles calculations, Chem. Eur. J. **20**, 15188–15196 (2014).

[17] C. Martineau, M. Allix, M. R. Suchomel, F. Porcher, F. Vivet, C. Legein, M. Body, D. Massiot, F. Taulelle, and F. Fayon, Structure determination of Ba_5AlF_{13} by coupling electron, synchrotron and neutron powder diffraction, solid-state NMR and ab initio calculations, Dalton Trans. **45**, 15565–15574 (2016).

[18] A. Rakhmatullin, I. B. Polovov, D. Maltsev, M. Allix, V. Volkovich, A. V. Chukin, M. Boča, and C. Bessada, Combined approach for the structural characterization of alkali fluoroscandates: Solid-state NMR, powder X-ray diffraction, and density functional theory calculations, Inorg. Chem. **57**, 1184–1195 (2018).

[19] A. Rakhmatullin, M. Boča, J. Mlynáriková, E. Hadzimová, Z. Vasková, I. B. Polovov, and M. Mičušík, Solid state NMR and XPS of ternary fluorido-zirconates of various coordination modes, J. Fluor. Chem. **208**, 24–35 (2018).

[20] L. Martel, E. Capelli, M. Body, M. Klipfel, O. Beneš, L. Maksoud, P. E. Raison, E. Suard, L. Visscher, C. Bessada, C. Legein, T. Charpentier, and A. Kovács, Insight into the crystalline structure of ThF_4 with the combined use of neutron diffraction, ^{19}F magic-angle spinning-NMR, and density functional theory calculations, Inorg. Chem. **57**, 15350–15360 (2018).

[21] M. A. DeVore, C. A. Klug, M. R. Kriz, L. E. Roy, and M. S. Wellons, Investigations of uranyl fluoride sesquihydrate ($UO_2F_2{\cdot}1.57H_2O$): Combining ^{19}F solid-state MAS NMR spectroscopy and GIPAW chemical shift calculations, J. Phys. Chem. A **122**, 6873–6878 (2018).

[22] M. Albino, M. Body, C. Legein, A. Hémon-Ribaud, M. Leblanc, V. Maisonneuve, and J. Lhoste, NMR crystallography, hydrogen bonding and optical properties of the novel 2D hybrid oxyfluorotitanate $[H_2 taz]_2{\cdot}(Ti_5O_5F_{12})$, Crys. Grow. Des. **18**, 6873–6884 (2018).

[23] J. Dabachi, M. Body, J. Dittmer, A. Rakhmatullin, F. Fayon, and C. Legein, Insight into the factors influencing NMR parameters in crystalline materials from the KF-–YF_3 binary system, Dalton Trans. **48**, 587–601 (2019).

[24] A. P. Bartók and J. R. Yates, Ultrasoft pseudopotentials with kinetic energy density support: Implementing the Tran-Blaha potential, Phys. Rev. B **99**, 235103 (2019).

[25] M. Murakami, Y. Noda, and K. Takegoshi, Terminal and bridging fluorine ligands in TiF_4 as studied by ^{19}F NMR in solids, Sol. Stat. Nucl. Mag. Res. **101**, 82 (2019).

[26] F. Ding, K. J. Griffith, C. P. Koçer, R. J. Saballos, Y. Wang, C. Zhang, M. L. Nisbet, A. J. Morris, J. M. Rondinelli, and K. R. Poeppelmeier, Multimodal structure solution with ^{19}F NMR crystallography of spin singlet molybdenum oxyfluorides, J. Am. Chem. Soc. **142**, 12288 (2020).

[27] M. Albino, J. Lhoste, M. Body, C. Legein, A. Hémon-Ribaud, V. Maisonneuve, and M. Leblanc, Topotactic desolvation and condensation reactions of 3D $Zn_3TiF_7(H_2O)_2(taz)_3 \cdot S$ (s = $3\,H_2O$ or C_2H_5OH), Dalton Trans. **49**, 17758–17771 (2020).

[28] A. Rakhmatullin, M. S. Molokeev, G. King, I. B. Polovov, K. V. Maksimtsev, E. Chesneau, E. Suard, R. Bakirov, F. Šimko, C. Bessada, and M. Allix, Polymorphs of Rb_3ScF_6: X-ray and neutron diffraction, solid-state NMR, and density functional theory calculations study, Inorg. Chem. **60**, 6016–6026 (2021).

[29] C. Legein, M. Body, J. Lhoste, W. Li, T. Charpentier, and D. Dambournet, Synthesis, crystal structure and ^{19}F NMR parameters modelling of $CaTiF_6(H_2O)_2$ yielding to a revision of the bond-valence parameters for the Ti^{4+}/F^- ion pair, J. Sol. Stat. Chem. **319**, 123793 (2023).

[30] R. Laskowski and P. Blaha, Calculations of NMR chemical shifts with APW-based methods, Phys. Rev. B **85**, 035132 (2012).

[31] R. Laskowski, P. Blaha, and F. Tran, Assessment of DFT functionals with NMR chemical shifts, Phys. Rev. B **87**, 195130 (2013).

[32] G. A. de Wijs, R. Laskowski, P. Blaha, R. W. A. Havenith, G. Kresse, and M. Marsman, NMR shieldings from density functional perturbation theory: GIPAW versus all-electron calculations, J. Chem. Phys. **146**, 10.1063/1.4975122 (2017).

[33] G. Kresse and J. Furthmüller, Efficient iterative schemes for *ab initio* total-energy calculations using a plane-wave basis set, Phys. Rev. B **54**, 11169 (1996).

[34] G. Kresse and J. Furthmüller, Efficiency of ab-initio total energy calculations for metals and semiconductors using a plane-wave basis set, Comput. Mater. Sci. **6**, 15 (1996).

[35] S. Grimme, J. Antony, S. Ehrlich, and H. Krieg, A consistent and accurate *ab initio* parametrization of density functional dispersion correction (DFT-D) for the 94 elements H–Pu, J. Chem. Phys. **132**, 154104 (2010).

[36] B. J. Morgan, Data analysis for “Correlated Anion-Disorder in Heteroanionic Cubic $TiOF_2$” (2024), GitHub: `bjmorgan/data_TiOF2`.
